# Proprietary Medicines Containing *Bupleurum chinense* DC. (Chaihu) for Depression: Network Meta-Analysis and Network Pharmacology Prediction

**DOI:** 10.3389/fphar.2022.773537

**Published:** 2022-04-06

**Authors:** Qiao-feng Li, Wen-tian Lu, Qing Zhang, Yan-dong Zhao, Cheng-yu Wu, Hui-fang Zhou

**Affiliations:** ^1^ College of Traditional Chinese Medicine, College of Integrated Chinese and Western Medicine, Nanjing University of Chinese Medicine, Nanjing, China; ^2^ The First Clinical Medical College, Nanjing University of Chinese Medicine, Nanjing, China; ^3^ Department of Science and Technology, The Affiliated Hospital of Nanjing University of Chinese Medicine, Nanjing, China; ^4^ Department of Gynaecology, The Affiliated Hospital of Nanjing University of Chinese Medicine, Nanjing, China

**Keywords:** randomized controlled trials, *Bupleurum chinense* DC., Chaihu, proprietary Chinese medicines, depression, network meta-analysis, network pharmacology

## Abstract

**Background and Aims:** The rapid development of society has resulted in great competitive pressures, leading to the increase in suicide rates as well as incidence and recurrence of depression in recent years. Proprietary Chinese medicines containing *Bupleurum chinense* DC. (Chaihu) are widely used in clinical practice. This study aimed at evaluating the efficacy and safety of oral proprietary Chinese medicines containing Chaihu for treating depression by network meta-analysis (NMA) and exploring the potential pharmacological mechanisms of the optimal drugs obtained based on NMA.

**Methods:** This study searched for clinical randomized controlled trial studies (RCTs) about Chaihu-containing products alone or in combination with selective serotonin reuptake inhibitors (SSRI), serotonin-norepinephrine reuptake inhibitors (SNRI), and cyclic antidepressants (CAS) for depression in eight databases. The search deadline is from data inception to April 2021. For efficacy assessment, the clinical response rate, the Hamilton Depression Scale-17 (HAMD-17), and adverse reactions were calculated. The methodological quality of the included studies was assessed for risk of bias following the *Cochrane Handbook for Systematic Reviews* of *Interventions*, and the data were subjected to NMA via the Stata version 16.0 software. Subsequently, the optimal drug obtained from the NMA results, Danzhi Xiaoyao pill (DZXY), was used to conduct network pharmacology analysis. We searched databases to acquire bioactive and potential targets of DZXY and depression-related targets. The protein-protein interaction (PPI) network, component-target network, the Gene Ontology (GO), and the Kyoto Encyclopedia of Genes and Genomes (KEGG) analysis were performed by the STRING database, Cytoscape 3.9.0 software, and R version 4.1.2, respectively.

**Results:** Thirty-seven RCTs, with a total of 3,263 patients, involving seven oral proprietary Chinese medicines containing Chaihu, were finally included. The results of the NMA demonstrated that the top four interventions with the best efficiency were Jiawei Xiaoyao + SSRI, DZXY + SNRI, Xiaoyao pill + SSRI, and Jieyu pill + SNRI; the top four interventions reducing HAMD score were DZXY + SNRI, Jiawei Xiaoyao, Jieyu pill, and Puyu pill + SNRI; the top four interventions with the least adverse effects were Jieyu pill, Anle pill + SSRI, DZXY + SNRI, and Puyu pill + SNRI. In the aspects above, DZXY + SNRI performed better than other treatments. After network meta-analysis, we conducted a network pharmacology-based strategy on the optimal drugs, DZXY, to provide the pharmacological basis for a conclusion. A total of 147 active compounds and 248 targets in DZXY were identified, of which 175 overlapping targets related to depression. Bioinformatics analysis revealed that MAPK3, JUN, MAPK14, MYC, MAPK1, etc. could become potential therapeutic targets. The MAPK signaling pathway might play an essential role in DZXY against depression.

**Conclusion:** This is the very first systematic review and network meta-analysis evaluating different oral proprietary Chinese medicines containing Chaihu in depressive disorder. This study suggested that the combination of proprietary Chinese medicines containing Chaihu with antidepressants was generally better than antidepressant treatment. The incidence of adverse reactions with antidepressants alone was higher than that with proprietary Chinese medicines containing Chaihu alone or in combination with antidepressants. DZXY + SNRI showed significantly better results in efficacy, HAMD scores, and safety. The antidepressant effect of DZXY may be related to its regulation of neuroinflammation and apoptosis.

## Introduction

Depression is a psychiatric disorder with a high prevalence. A new report published by the World Health Organization (WHO) in 2017, *Depression and Other Common Mental Disorders: Global Health Estimates*, states that the number of people with depression increased by 18.4% globally between 2005 and 2015, approximately 322 million people, accounting for 4.4% of the world’s population, with a higher prevalence of major depressive disorder (MDD) in women than in men (5.1 vs. 3.6%) (Friedrich, 2017; World Health Organization, 2017). The global prevalence of MDD is approximately 4.7% (Ferrari et al., 2013a). Depression is the second leading cause of year lost due to disability (YLD), leads to illness-induced disability, and is an important risk factor for suicide, with more than 800,000 suicides per year. MDD accounts for 8.2% of global YLDs. Depression is the leading cause of disability-adjusted life year (DALY); MDD accounts for 2.5% (1.9%–3.2%) and poor mood accounts for 0.5% (0.3%–0.6%) of global DALYs (Ferrari et al., 2013b). The incidence of depression, relapse rate, and suicide rate are growing year by year as socialist modernization and competitive pressure accelerate, putting a heavy burden on society, families, and individuals. Depression has gradually become the focus of research in psychiatric diseases worldwide. Thus, there is an urgent need to discover efficient and safe treatment modalities to improve the poor prognosis of MDD. Currently, antidepressants work mainly by inhibiting the re-setting of monoamines by high-affinity transporters in the brain. However, their effectiveness has long been questioned due to the slow onset of action, limited efficacy, side effects, and drug resistance in nearly one-third of depressed patients (Orrico-Sanchez et al., 2020). A meta-analysis of 21 antidepressants (including all second-generation antidepressants, two tricyclic antidepressants, trazodone, and nefazodone) summarized the available evidence on clinical effectiveness reporting that the efficacy of antidepressants compared with placebo is mostly nonsignificant and that the long-term balance of efficacy and safety remains understudied (Cipriani et al., 2018).

Depression is considered to be located in the liver and related to the heart and spleen and caused by the loss of liver distribution, spleen transportation, and heart nourishment from the perspective of traditional Chinese medicine (Sun et al., 2017). It is believed that traditional Chinese medicine (TCM) has the effect of regulating neuroplasticity and reducing neuroinflammation with major advantages of synergy and attenuation (Wang et al., 2016). *Bupleurum chinense* DC. (Chaihu) is the most often utilized in the sutra formulas for treating depression, except for *Glycyrrhiza uralensis* Fisch. ex DC. and *Ziziphus jujuba* Mill. (Zou et al., 2017). The clinical practice is mainly based on the formulas containing Chaihu to detoxify the liver and relieve depression (Zhao et al., 2015; Gao et al., 2018). In terms of meridians, Chaihu enters the liver, gallbladder, heart, triple warmer, stomach, and large intestine channels, and is a key botanical drug for relieving MDD symptoms. Discussing the pharmacological effects and mechanisms, Saikosaponin a in Chaihu can regulate the expression of NF-κB, MAPK and other signaling pathways as well as various inflammatory factors, and has various pharmacological activities, such as being antidepressant, antiatherosclerotic, anti-inflammatory, anti-tumor, anti-epileptic, and hepatoprotective (Zhang, 2014; Li et al., 2019; Liu et al., 2019; Cheng et al., 2021). Many studies have shown that the *Bupleurum chinense* DC. (Chaihu)- *Paeonia lactiflora* Pall. (Baishao) pair plays an important role in antidepressant treatment, significantly improving depression-like symptoms of chronic unpredictable mild stress-treated mice, and 25 depression-related biomarkers were analyzed and identified by liquid chromatography-tandem mass spectrometry (LC-MS), 16 of which were significantly modulated by the Chaihu-Bashao pair (Chen et al., 2020), and mainly involved in the positive regulation of neuronal apoptosis, response to estradiol and cobalt ions, ligand-free exogenous apoptotic signaling pathway and transcription initiation of RNA polymerase II promoter, and G protein-coupled acetylcholine receptor activity (Zhang et al., 2016; Lam et al., 2018; Ziegler and Domschke, 2018; Chang et al., 2020). The integrative nature of botanical drugs is marked by multi-component, multi-target, and multi-systemic effects that act together to achieve the desired outcome. This network pattern has challenged researchers to describe their exact mechanisms of action. Network pharmacology has been widely used to research botanical drugs, covering pharmacology, phytochemistry, and pharmacokinetics. Network pharmacology is a flexible method that combines bioinformatics, computational analysis, and polypharmacology and can be used to explore the complex interactions in botanical drugs. (Zhang et al., 2019). Therefore, this study systematically evaluated and screened out Chaihu-containing products better in terms of efficacy and safety and explored their potential targets and pathways of action based on network pharmacology analysis.

## Methods of Network Meta-Analysis

### Inclusion Criteria

We included the RCTs with the following criteria: 1) Type of clinical research: Clinical randomized controlled trial (RCT), languages include Chinese and English; 2) Subjects: Patients who were clearly diagnosed with depression, regardless of age, gender, or race, whose diagnosis was mainly based on the relevant criteria in the Chinese Classification of Mental Disorders Third Edition (CCMD-3) (Psychosis Branch of Chinese Medical Association, 2001), the Diagnostic and Statistical Manual of Mental Disorders (DSM-IV) (American Psychiatric Association, 2013), or the International Statistical Classification of Diseases and Related Health Problems 10^th^ Revision (ICD-10) (World Health organization, 1992); 3) Interventions: The test group was treated with proprietary Chinese medicine containing Chaihu, approved for marketing by the National Regulatory Authority, and whose functional subject was the treatment of hepatic depression, or in combination with selective serotonin reuptake inhibitor (SSRI), serotonin-norepinephrine reuptake inhibitor (SNRI), cyclic antidepressants (CAS), or placebo; the control group was treated with SSRI, SNRI, CAS or placebo; the duration of treatment is not limited, but must include the fourth week as the node; 4) Outcome indicators: clinical efficiency, Hamilton Depression Rating Scale—17 (HAMD-17), adverse effects.

### Exclusion Criteria

Drug-induced secondary depression, somatic disease-induced secondary depression, psychiatric disease-induced secondary depressive symptoms, postpartum depression, menopausal depression, or insomnia-induced depression; inaccessibility of the entire text, or missing data; utilization of HAMD-21 or HAMD-24; interventions combined with other proprietary Chinese medicine, tonics, or transcranial magnetic stimulation treatment; duplication of published literature.

### Data Sources and Searches

We identified eligible trials by searching PubMed, Web of Science, The Cochrane Library, Embase, and the China National Knowledge Infrastructure (CNKI), Wanfang database, VIP database, and China Biology Medicine disc (CBM disc). The search with subject limitations within the English or Chinese language was conducted between the inception of each database and April 2020. The detailed search strategy is listed in Supplementary Table S1.

### Data Extraction and Quality Assessment

All of the obtained literature were imported into NoteExpress software for screening and duplicate literature was eliminated. Then two independent assessors screened, evaluated, and extracted data of every trial included in our review according to the inclusion and exclusion criteria. The extracts contained the article title, author of the literature, year, sample content, baseline status, interventions, outcome indicators, and duration of treatment. Discrepancies were resolved by negotiation or an authoritative third party.

The methodological quality of the included studies was assessed for risk of bias according to the quality assessment criteria in the Cochrane Handbook for Systematic Reviews of Interventions, version 5.10.

### Data Synthesis

We used Stata version 16.0 software for data analysis. Relative risk (RR) was used for count data, weighted mean difference (WMD) was used for measurement data, and 95% confidence interval (CI) was used for interval estimation as an indicator of effect size. When the data extracted from the trials were brought into the Stata version 16.0 software for calculation, the results of direct comparisons were compared with those of indirect comparisons using the node-splitting model in the software to observe whether the results were consistent, and then the results of the consistency test were clarified. If there was no statistical difference (*p* > 0.05), the consistency model was used to perform a network meta-analysis of the efficacy of various drugs for depression; if there was a statistical difference (*p* < 0.05), a specific analysis of the sources of non-consistency was performed. After comparing the therapeutic effects of various drugs, a ranked probability ranking chart was used to evaluate the advantages and disadvantages of each drug and to assess the likelihood of each drug being the best treatment.

## Methods of Network Pharmacology Analysis

### Data Preparation

The compounds of nine botanical drugs [*Bupleurum chinense* DC. (Apiaceae; *Bupleuri* radix), *Angelica sinensis* (Oliv.) Diels (Apiaceae; *Angelicae sinensis* radix), *Paeonia lactiflora* Pall. (Paeoniaceae; *Paeoniae* radix alba), *Atractylodes macrocephala* Koidz. (Asteraceae; *Atractylodis macrocephalae* rhizoma), *Poria cocos* (Schw.) Wolf. (Polyporaceae; Poria), *Glycyrrhiza uralensis* Fisch. ex DC. (Fabaceae; *Glycyrrhizae* radix et rhizoma), *Paeonia* × suffruticosa Andrews (Paeoniaceae; *Moutan* cortex), *Gardenia jasminoides* J. Ellis (Rubiaceae; *Gardeniae* fructus), *Mentha canadensis* L. (Lamiaceae; *Menthae haplocalycis* herba)] in DZXY were obtained from Traditional Chinese medicine systems pharmacology database and analysis platform (TCMSP, https://old.tcmsp-e.com/tcmsp.php), which can provide information of ingredients of botanical drugs and have been widely reported to be available for compounds exploration in network pharmacology (Ru et al., 2014; Liu et al., 2016; Xu et al., 2019; Chen, 2011). Subsequently, according to the ADME criteria (i.e., oral bioavailability (OB)≥30% and drug-likeness (DL)≥0.18,), active compounds of DZXY were retained for further research. OB and DL were one of the most significant pharmacokinetic parameters of the ADME (absorption, distribution, metabolism, and excretion) properties of drugs (Tian et al., 2012). In addition, depression-related targets were retrieved by searching the keyword “depression” from four public databases:, namelyDrugBank (https://www.drugbank.ca/), TTD (http://db.idrblab.net/ttd), GeneCards (https://www.genecards.org/, Relevance score ≥1), and OMIM (http://omim.org/), all of which can be applied to collect disease-related targets (Stelzer et al., 2016; Amberger and Hamosh, 2017; Wishart et al., 2018; Wang et al., 2020; Zhou et al., 2022). Next, confirmed human genes were downloaded from the Uniprot database (https://www.uniprot.org/) and protein target names were converted to their corresponding official gene symbols by PERL software.

### Protein-Protein Interaction and Enrichment Analysis

To further identify the core regulatory targets, The protein-protein interaction (PPI) analysis was performed by submitting the overlapping targets of active compounds to the STRING database (https://string-db.org/), which currently contains the largest number of organisms and proteins, as well as broad and diverse benchmarked data sources (Szklarczyk et al., 2019). The species was limited to *Homo sapiens*, the minimum required interaction score was set to 0.900, and the independent target protein nodes were hidden. Subsequently, the tsv file of PPI results was exported from STRING and imported into Cytoscape 3.9.0. The CytoNCA plugin in Cytoscape software was utilized to calculate six parameters: betweenness centrality (BC), closeness centrality (CC), degree centrality (DC), eigenvector centrality (EC), network centrality (NC), and local average connectivity (LAC). Nodes with all six parameters higher than the corresponding median values were retained to construct the core PPI. Another plugin, CytoHubba, was used to identify hub genes (Chin et al., 2014). We used the maximal clique centrality (MCC) algorithm to rank the nodes for network centrality and select the top 16 as candidate genes.

The Gene Ontology (GO) enrichment analysis and Kyoto Encyclopedia of Genes and Genomes (KEGG) pathway enrichment analysis were conducted to further study the functions of the potential depression-resistant target genes of DZXY based on R version 4.1.2. Only functional terms and pathways with q values < 0.05 were considered statistically significant and retained.

### Construction of Related Networks

Data of DZXY-depression targets, bioactive compounds, disease, botanical drugs, or signaling pathways were imported into the Cytoscape 3.9.0 software. After being polished, three networks were constructed: (1) Component-target network of treatment with DZXY for depression; and (2) PPI network of treatment with DZXY for depression.

## Results of Network Meta-Analysis

### Study Screening

**FIGURE 1:**
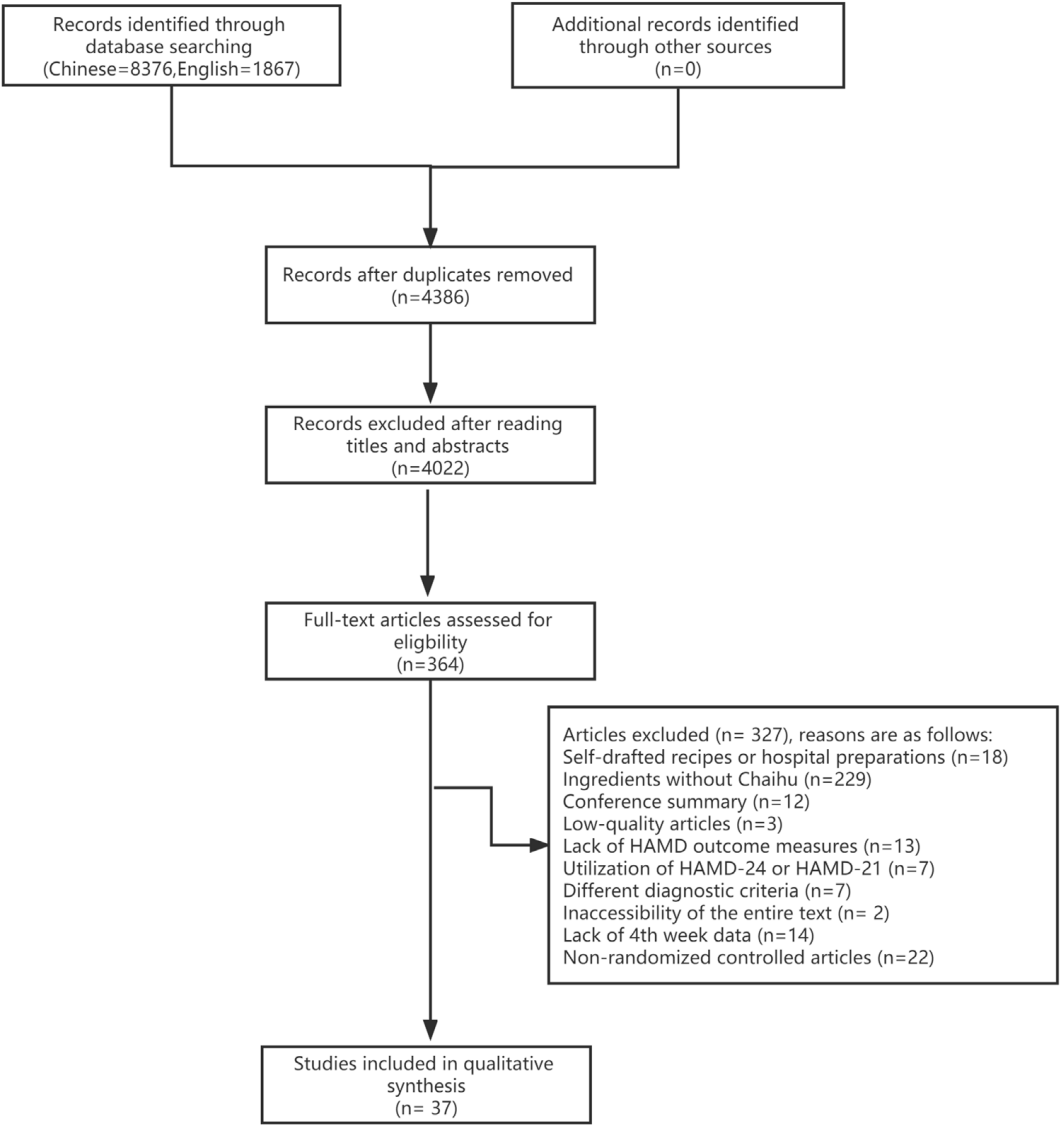
Literature screening process.

A total of 10,243 relevant original literature were detected in this network meta-analysis, including 1,867 English literature and 8,376 Chinese literature involving 13 drug regimens (SNRI, CAS, Anle pill + SSRI, Danzhi Xiaoyao pill + SNRI, Jiawei Xiaoyao pill, Jiawei Xiaoyao pill + CAS, Jiawei Xiaoyao + SSRI, Jieyu pill, Jieyu pill + SNRI, Puyu pill + SNRI, Shumian capsule + SSRI, Xiaoyao pill + CAS, and Xiaoyao pill + SSRI). After reading titles and abstracts, we excluded 4,022 studies; 37 of the 364 remaining studies satisfied inclusion criteria and were thus included for qualitative synthesis, as shown in Figure 1 **.**

### Characteristics and Quality of Study

**FIGURE 2:**
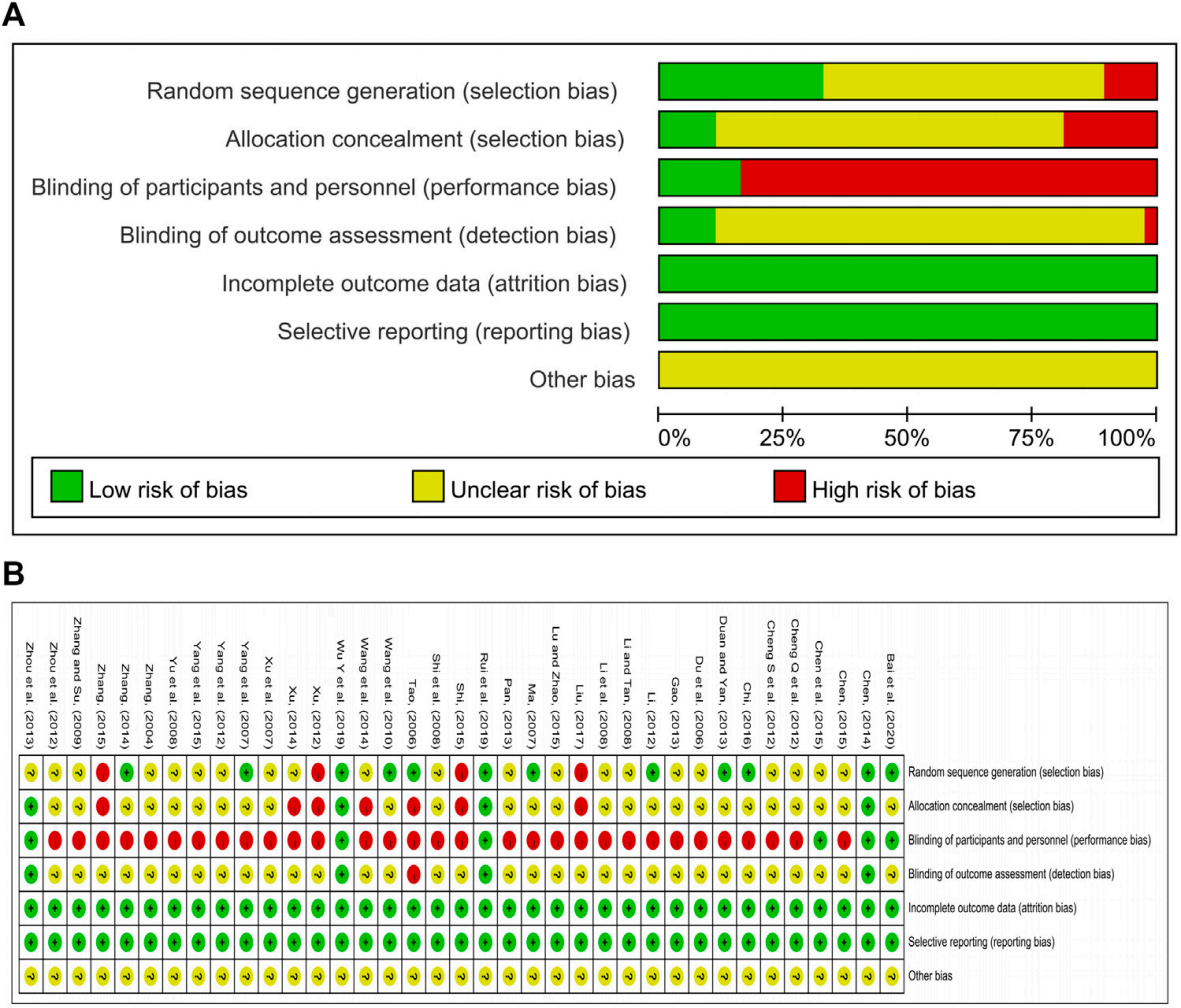
Risk of bias graph **(A)** and risk of bias summary **(B)**, respectively.

Thirty-seven RCTs with a total of 3,263 patients were included, involving 7 oral proprietary Chinese medicines containing Chaihu (Anle pill, Danzhi Xiaoyao pill, Jiawei Xiaoyao pill, Jieyu pill, Puyu pill, Shumian capsule, and Xiaoyao pill), as well as 13 interventions, mainly proprietary Chinese medicines containing Chaihu alone or in combination with three classes of antidepressant western medicines (SSRI, SNRI, and CAS). Twenty-eight trials discussed the efficacy of the oral proprietary Chinese medicines containing Chaihu for treating depression, 37 trials reported HAMD-17 scores, and 32 trials assessed adverse effects in patients after treatment. There were no statistical differences at the baseline between the groups. The basic characteristics of the included studies are shown in Table 1. The results of the risk of bias evaluation of the included studies are shown in Figure 2. The ingredients of proprietary medicines used in 37 studies are shown in Supplementary Table S1.

**TABLE 1:** Basic information of included studies.

Study	Sample (male/female)		Average age/years		Course/months		Interventions		Treatment/weeks	Outcomes
	T	C	T	C	T	C	T	C		
Wu Y et al. (2019)	78 (−/−)	62 (−/−)	—	—	—	—	Jiawei Xiaoyao + SSRI	SSRI + placebo	4	①②③
Bai et al. (2020)	45 (13/32)	45 (12/33)	41.73 ± 7.86	43.56 ± 8.03	—	—	Jieyu pill + SNRI	SNRI	8	①②③
Su et al. (2019)	95 (27/68)	96 (29/67)	42 ± 12	44 ± 13	—	—	Jiawei Xiaoyao + placebo	SSRI + placebo	8	①②③
Chi, (2016)	66 (25/41)	63 (25/38)	45.24 ± 10.85	45.36 ± 11.13	(2.71 ± 0.84) years	(2.75 ± 0.86) years	Xiaoyao pill + SSRI	SSRI	4	②③
Zhang, (2014)	58 (33/25)	58 (32/26)	44.9 ± 12.6	45.2 ± 12.9	(2.1 ± 0.8)years	(2.2 ± 0.8) years	Jiawei Xiaoyao + SSRI	SSRI	4	①②③
Xu, (2014)	60 (25/35)	60 (28/32)	37.31 ± 9.46	36.66 ± 8.48	(0.9 ± 0.6) years	(0.8 ± 1.3) years	Xiaoyao pill + SNRI	SNRI	8	①②③
Wang et al. (2014)	80 (37/43)	80 (39/41)	38.7 ± 5.2	37.5 ± 4.8	8.1 ± 1.9	7.8 ± 2.3	Jieyu pill	SNRI	8	①②③
Duan and Yan, (2013)	30 (20/10)	30 (10/20)	37 ± 10	36 ± 14; 36 ± 11	9.0 ± 1.0	8.0 ± 1.0	Jieyu pill + SNRI	SNRI	6	①②③
Pan, (2013)	47 (28/19)	47 (27/20)	67.4 ± 6.3	67.8 ± 6.1	5.3 ± 3.1	5.2 ± 2.9	Jieyu pill + SSRI	SSRI	8	①②③
Li, (2012)	30 (10/20)	30 (11/19)	37 ± 12	36 ± 12	8.0 ± 2.0	9.0 ± 2.0	Jieyu pill + SSRI	SSRI	8	②③
Zhou et al. (2013)	65 (−/−)	62 (−/−)	—	—	—	—	Jiawei Xiaoyao + placebo	SSRI + placebo	8	①②③
Chen, (2014)	34 (8/26)	31 (12/19)	—	—	—	—	Jiawei Xiaoyao pill + placebo	SSRI + placebo	8	①②③
Zhang and Su, (2009)	31 (15/16)	31 (17/14)	36 ± 7.41	38 ± 5.22	4.12 ± 3.2	4.31 ± 3.1	Puyu + SNRI	SNRI	6	①②③
Yu et al. (2008)	49 (19/30)	49 (20/29)	36.12 ± 10.37	36.58 ± 10.65	(3.05 ± 3.68) years	(3.25 ± 3.85)years	Anle pill + SNRI	SNRI	6	②
Ma, (2007)	30 (14/16)	30 (16/14)	35.65 ± 7.25	35.29 ± 5.80	(4.55 ± 3.90) years	(4.56 ± 4.00)years	Xiaoyao pill + SSRI	SSRI	8	①②③
Du et al. (2006)	50 (23/27)	50 (24/26)	36.18 ± 10.47	37.28 ± 10.75	(3.08土3.59) years	(3.58 ± 3.8) years	Puyu + SNRI	SNRI	6	①②③
Zhang, (2004)	25 (15/10)	25 (9/16)	38.0 ± 11.9	43.0 ± 11.4	—	—	Xiaoyao pill + SSRI	SSRI	6	②③
Yang et al. (2007)	32 (9/23)	32 (11/21)	32 ± 11	34 ± 17	(2.5 ± 4.8) weeks	(3.2 ± 5.2) weeks	Jiawei Xiaoyao pill + TcAs	SSRI	12	①②③
Wang et al. (2010)	38 (21/17)	36 (19/17)	35.8 ± 8.6	33.5 ± 9.1	15.7 ± 10.2	14.2 ± 11.4	Jieyu pill + SSRI	SSRI	6	①②③
Tao, (2006)	41 (18/23)	45 (19/26)	34 ± 13	31 ± 9	(7.43 ± 8.07) weeks	(8.88 ± 9.82) weeks	Jieyu pill	SNRI	6	②
Li et al. (2008)	29 (15/14)	28 (14/14)	65.1 ± 6.3	65.5 ± 7.1	(9.5 ± 2.3) years	(10.3 ± 6.0) years	Jieyu pill	CAS	6	①②③
Liu, (2017)	30 (15/15)	30 (14/16)	34 ± 4.4	36 ± 3.1	(0.8 ± 0.6) years	(0.8 ± 1.1) years	Xiaoyao pill + SSRI	SSRI	6	②③
Shi, (2015)	60 (36/24)	60 (−/−)	32 ± 2.3	—	11.3 ± 2.6	—	Anle pill + SSRI	SSRI	8	①②③
Zhang, (2015)	56 (−/−)	56 (−/−)	—	—	—	—	Xiaoyao pill + SSRI	SSRI	6	①②
Chen, (2015)	23 (16/7)	21 (16/5)	37.75 ± 11.56	36.75 ± 12.52	13.26 ± 11.75	14.17 ± 13.25	Danzhi Xiaoyao pill + SNRI	SNRI	4	①②③
Cheng Q et al. (2012)	31 (15/16)	31 (14/17)	43.45 ± 11.27	45.36 ± 12.38	6.9 ± 2.6	7.1 ± 1.8	Shumian capsule + SSRI	SSRI	8	②③
Chen et al. (2015)	34 (13/21)	34 (12/22)	35.00 ± 6.25	34.31 ± 7.12	(3.52 ± 2.17) years	(3.23 ± 2.08) years	Shumian capsule + SSRI	SSRI	8	①②③
Cheng S et al. (2012)	40 (22/18)	40 (23/17)	18.2 ± 2.5	17.6 ± 2.2	5.1 ± 4.2	5.5 ± 4.5	Shumian capsule + SSRI	SSRI	6	①②
Xu, (2012)	30 (12/18)	30 (14/16)	36.9 ± 11.2	38.2 ± 11.5	—	—	Jieyu pill	SSRI	6	①②③
Shi et al. (2008)	68 (29/39)	67 (31/36)	37.9 ± 11.5	39.5 ± 13.1	—	—	Jieyu pill	SSRI	6	①②③
Yang et al. (2015)	36 (19/17)	32 (17/15)	—	—	—	—	Jieyu pill	CAS	6	①②③
Yang et al. (2012)	30 (16/14)	30 (17/13)	66．52 ± 4．21	65．31 ± 3．79	4．58 ± 3．12	4．73 ± 3．21	Jieyu pill + SSRI	SSRI	8	①②③
Xu et al. (2007)	22 (10/12)	19 (9/10)	24.51 ± 18.73	26.47 ± 17.81	8.23 ± 6.64	9.04 ± 8.12	Jieyu pill	SNRI	6	①②③
Lu and Zhao, (2015)	55 (22/23)	55 (26/29)	37.3 ± 9.45	36.68 ± 7.46	(0.9 ± 0.6) years	(0.8 ± 1.2) years	Jiawei Xiaoyao pill + SSRI	SSRI	8	①②③
Li and Tan, (2008)	30 (14/16)	30 (11/19)	39.3 ± 14.8	4.7 ± 5.8	38.9 ± 15.2	4.7 ± 5.6	Xiaoyao pill + TcAs	CAS	8	①②③
Zhou et al. (2012)	63 (25/38)	63 (28/35)	37.32 ± 9.466	36.68 ± 8.486	(2.5 ± 2.3) years	(2.8 ± 3.9) years	Xiaoyao pill + SSRI	SSRI	8	②③
Gao, (2013)	40 (23/17)	36 (20/16)	28.6 ± 5.8	27.4 ± 6.3	(5.5 ± 5.9) years	(5.7 ± 6.2) years	Xiaoyao pill + SSRI	SSRI	8	①②③

T, treatment group; C, control group; —, not mentioned; SSRI, selective serotonin reuptake inhibitor; SNRI, serotonin-norepinephrine reuptake inhibitor; CAS, cyclic antidepressants; ①, effective rate; ②, Hamilton Depression Rating Scale (HAMD) score; ③, adverse reaction.

### Total Effective Rate

**FIGURE 3:**
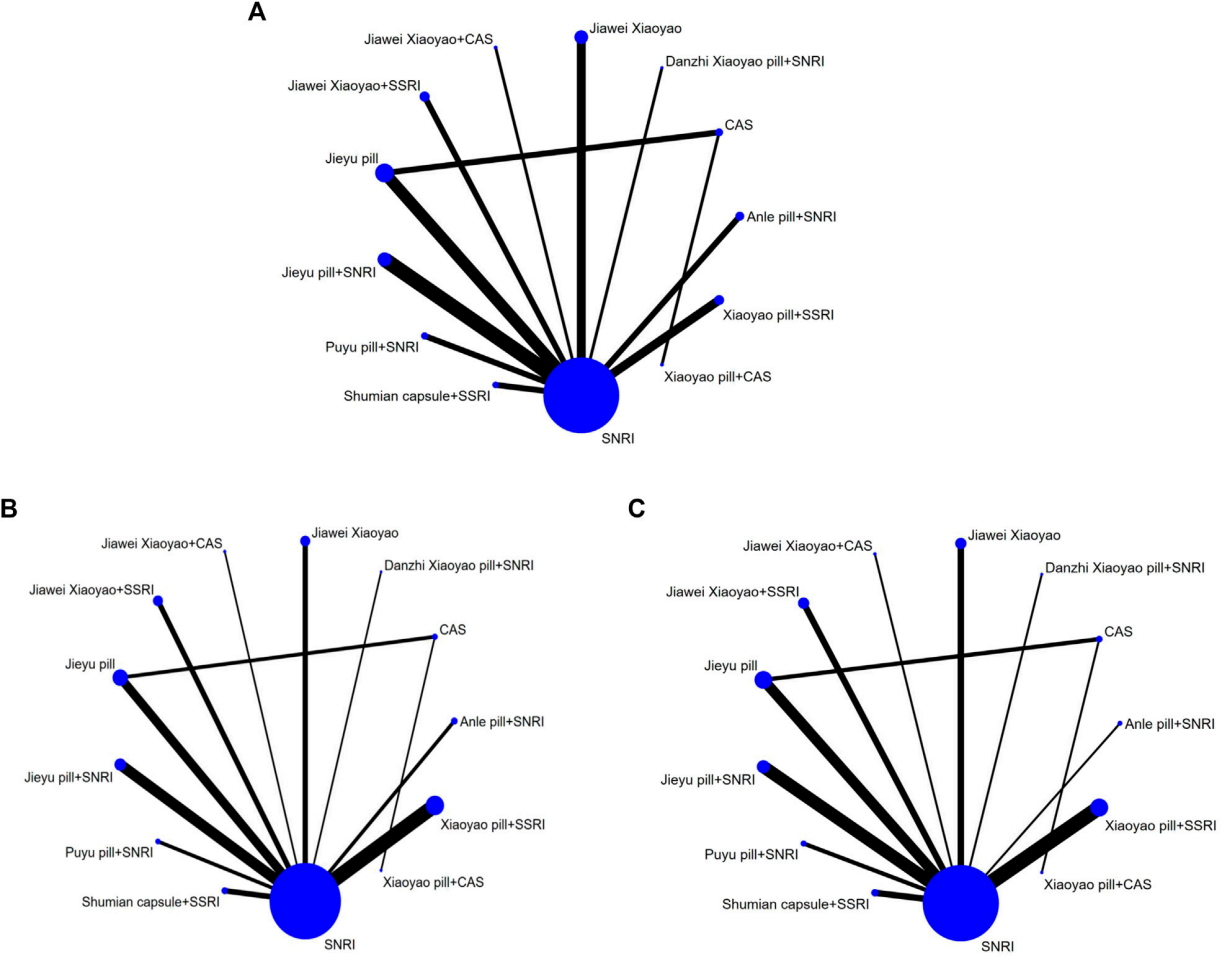
Evidence network of **(A)** total effective rate, **(B)** HAMD, and **(C)** adverse reactions.

The results of the evidence network revealed the following: twenty-eight studies reported the efficiency of different medications for depression involving 13 dosing regimens. The vertices in the network evidence map represent different intervention methods, the size of the vertices represents the sample size included in each intervention method, the lines between the vertices indicate the direct comparison existing between two intervention methods, and the thickness of the lines is proportional to the number of relevant studies. Direct or indirect evidence existed between different intervention methods, and the basic conditions for network meta-analysis (NMA) were present (Figure 3A).

The results of direct and indirect comparisons in the NMA were tested for consistency (*p* > 0.05), indicating that the overall consistency between direct and indirect comparisons was good, so the consistency model analysis was applied. The results of the NMA showed that among 78 two-by-two comparisons, a total of nine comparisons had statistically significant differences. The efficiency of CAS treatment was lower than that of Jiawei Xiaoyao pill + SSRI [RR = 0.80, 95% CI (0.65, 0.98)]; the efficiency of SNRI treatment was lower than that of Jiawei Xiaoyao + SSRI [RR = 0.78, 95%CI (0.68, 0.90)]; the efficiency of Jiawei Xiaoyao pill treatment was lower than that of Jiawei Xiaoyao + SSRI [RR = 0.78, 95%CI (0.66, 0.92)]; the efficiency of Jiawei Xiaoyao + CAS treatment was lower than that of Jiawei Xiaoyao pill + SSRI [RR = 0.75, 95%CI (0.62, 0.92)]. The effective rate of Jiawei Xiaoyao pill + SSRI was higher than that of the Jieyu pill [RR = 1.29, 95%CI (1.10, 1.51)], Jieyu pill + SNRI [RR = 1.20, 95%CI (1.03, 1.40)], Puyu pill + SNRI [RR = 1.28, 95%CI (1.10, 1.49)], Shumian Capsule + SSRI [RR = 1.28, 95%CI (1.09, 1.49)], and Xiaoyao pill + SSRI [RR = 1.20, 95%CI (1.03, 1.41)]. The rest of the two comparisons were not statistically significant. Thus, it can be seen that the effect advantage of treatment with Jiawei Xiaoyao pill + SSRI is more prominent (Table 2).

**TABLE 2:** Network meta-analysis of total effective rate RR (95%CI).

Interventions	CAS	SNRI	Anle pil + SSRI	Danzhi Xiaoyao pill + SNRI	Jiawei Xiaoyao	Jiawei Xiaoyao + CAS
CAS	0					
SNRI	1.02 (0.88, 1.18)	0				
Anle pill + SSRI	1.03 (0.87, 1.21)	1.01 (0.93, 1.08)	0			
Danzhi Xiaoyao pill + SNRI	0.82 (0.56, 1.21)	0.81 (0.57, 1.15)	0.80 (0.56, 1.15)	0		
Jiawei Xiaoyao	1.03 (0.87, 1.21)	1.00 (0.92, 1.09)	1.00 (0.89, 1.12)	1.24 (0.86, 1.79)	0	
Jiawei Xiaoyao + CAS	1.06 (0.86, 1.29)	1.03 (0.90, 1.19)	1.03 (0.87, 1.21)	1.28 (0.87, 1.88)	1.03 (0.87, 1.22)	0
Jiawei Xiaoyao + SSRI	0.80 (0.65, 0.98)*	0.78 (0.68, 0.90)	0.78 (0.66, 0.91)	0.97 (0.66, 1.42)	0.78 (0.66, 0.92)*	0.75 (0.62, 0.92)*
Jieyu pill	1.03 (0.91, 1.16)	1.01 (0.93, 1.09)	1.00 (0.90, 1.11)	1.25 (0.87, 1.79)	1.00 (0.89, 1.12)	0.97 (0.83, 1.14)
Jieyu pill + SNRI	0.96 (0.82, 1.12)	0.94 (0.88, 1.00)	0.93 (0.84, 1.03)	1.16 (0.81, 1.67)	0.93 (0.84, 1.04)	0.91 (0.77, 1.06)
Puyu + SNRI	1.02 (0.88, 1.19)	1.00 (0.95, 1.06)	0.99 (0.91, 1.09)	1.24 (0.87, 1.78)	1.00 (0.90, 1.10)	0.97 (0.83, 1.13)
Shumian capsule + SSRI	1.02 (0.87, 1.19)	1.00 (0.93, 1.06)	0.99 (0.90, 1.10)	1.23 (0.86, 1.77)	0.99 (0.89, 1.10)	0.96 (0.82, 1.13)
Xiaoyao pill + CAS	0.99 (0.81, 1.22)	0.97 (0.76, 1.25)	0.97 (0.75, 1.25)	1.21 (0.78, 1.86)	0.97 (0.75, 1.26)	0.94 (0.71, 1.25)
Xiaoyao pill + SSRI	0.96 (0.82, 1.12)	0.94 (0.87, 1.00)	0.93 (0.84, 1.03)	1.16 (0.81, 1.67)	0.93 (0.84, 1.04)	0.91 (0.77, 1.06)

Interventions	Jiawei Xiaoyao + SSRI	Jieyu pill	Jieyu pill + SNRI	Puyu + SNRI	Shumian capsule + SSRI	Xiaoyao pill + CAS	Xiaoyao pill + SSRI
Jiawei Xiaoyao + SSRI	0						
Jieyu pill	1.29 (1.10, 1.51)*	0					
Jieyu pill + SNRI	1.20 (1.03, 1.40)*	0.93 (0.84, 1.03)	0				
Puyu + SNRI	1.28 (1.10, 1.49)*	0.99 (0.90, 1.09)	1.07 (0.98, 1.16)	0			
Shumian capsule + SSRI	1.28 (1.09, 1.49)*	0.99 (0.89, 1.10)	1.06 (0.97, 1.17)	1.00 (0.91, 1.09)	0		
Xiaoyao pill + CAS	1.25 (0.94, 1.66)	0.97 (0.76, 1.23)	1.04 (0.80, 1.34)	0.97 (0.76, 1.26)	0.98 (0.76, 1.26)	0	
Xiaoyao pill + SSRI	1.20 (1.03, 1.41)*	0.93 (0.84, 1.03)	1.00 (0.91, 1.10)	0.94 (0.86, 1.02)	0.94 (0.85, 1.04)	0.96 (0.74, 1.25)	0

The difference between the two groups was statistically significant^*^ *p*＜0.05 (same as Tables 5, 6).

We further ranked all treatments according to SUCRA. The results of the probability ranking of efficiency in treating depression were shown in Table 3. According to the SUCRA results, JiaWei Xiaoyao + SSRI, Danzhi Xiaoyao pill + SNRI, Xiaoyao pill+ SSRI, and Jieyu pill + SNRI were the top four with higher efficiency in the treatment of depression, where Jiawei Xiaoyao pill +SSRI may be the most effective intervention for the treatment of depression.

**TABLE 3:** Ranking of network meta-analysis of effective rate, HAMD, ADR.

Interventions	Effective rate				HAMD				ADR			
	SUCRA/%	PrBest	MeanRank	Rank	SUCRA/%	PrBest	MeanRank	Rank	SUCRA/%	PrBest	MeanRank	Rank
Jiawei Xiaoyao + SSRI	95.4	53.4	1.6	1	17.8	0.2	10.9	12	58.5	0	5.6	6
Danzhi Xiaoyao pill + SNRI	81.8	42.3	3.2	2	90.1	40.6	2.2	1	67.9	6.2	4.5	3
Xiaoyao pill + SSRI	70.5	0.2	4.5	3	63.7	0.4	5.4	5	62.9	0	5.1	5
Jieyu pill + SNRI	70.4	0.1	4.6	4	18.6	0	10.8	11	55.8	0	5.9	7
CAS	47.6	0.2	7.3	5	16.7	0	11	13	2.7	0.2	11.7	12
Xiaoyao pill + CAS	47.5	3.7	7.3	6	49.4	0.5	7.1	7	-	-	-	-
Shumian capsule + SSRI	38.5	0	8.4	7	46.5	1	7.4	8	42.1	0	7.4	8
SNRI	35.9	0	8.7	8	28	0	9.6	10	16.5	0	10.2	11
Puyu + SNRI	35.9	0	8.7	9	74.1	3.4	4.1	4	67.6	0.4	4.6	4
Jiawei Xiaoyao	34.9	0	8.8	10	79.1	31.9	3.5	2	42.1	0	7.4	8
Jieyu pill	33.5	0	9	11	75.5	7.1	3.9	3	99.1	90.5	1.1	1
Anle pill + SSRI	33.2	0	9	12	57.8	14.7	6.1	6	68.9	2.7	4.4	2
Jiawei Xiaoyao + CAS	24.9	0.1	10	13	32.7	0	9.1	9	21.8	0	9.6	10

**FIGURE 4:**
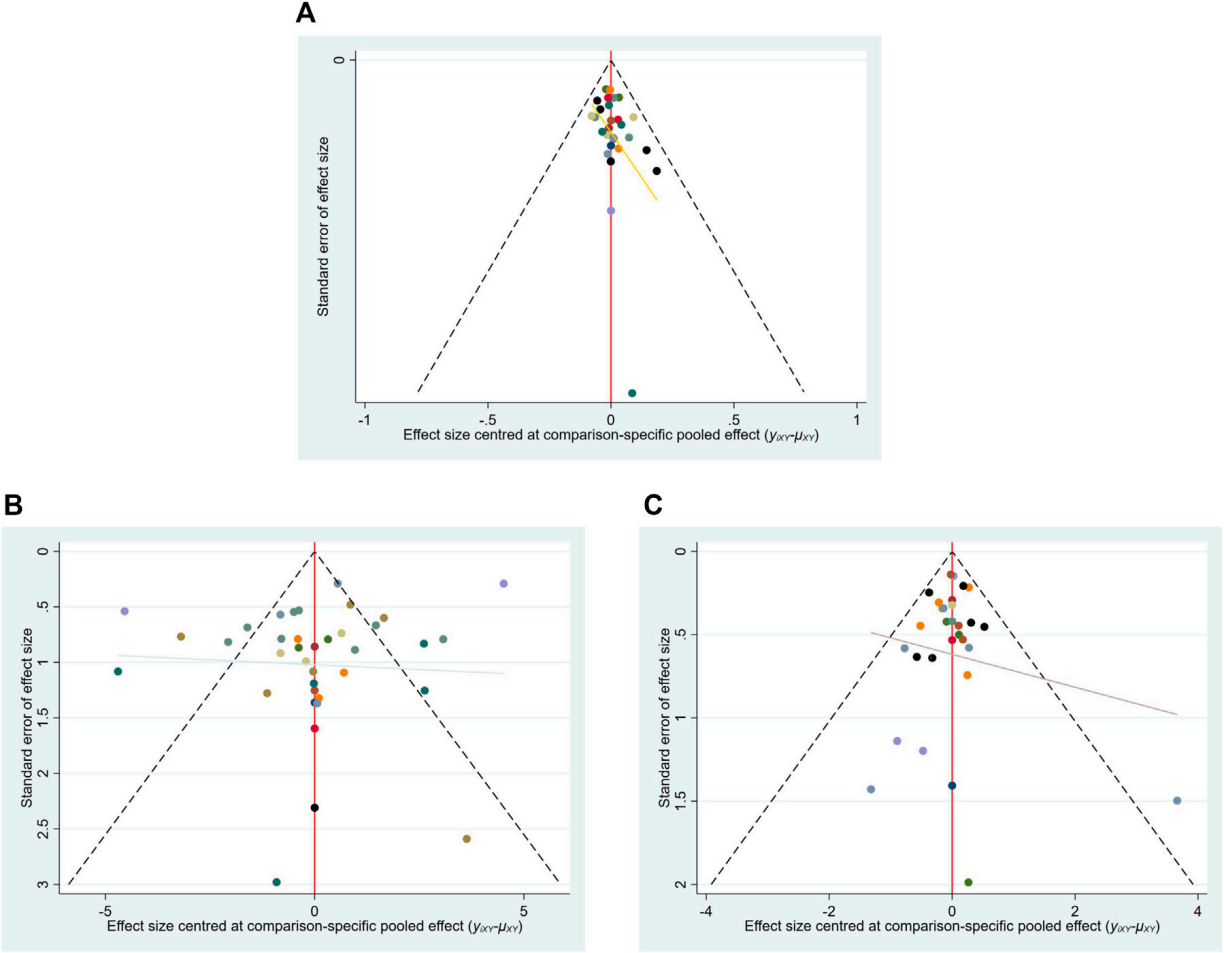
Funnel plot of **(A)** total effective rate, **(B)** HAMD, and **(C)** adverse reactions.

The funnel plot of the efficiency rate shows that the points are scattered and mostly symmetrical, suggesting that the possibility of a publication bias is minimal. And there is one scattered point located at the bottom of the funnel plot, indicating the influence of small sample sizes (Figure 4A).

### HAMD Scores

The results of the evidence network revealed the following: A total of 37 studies reported HAMD scores across 13 interventions, forming one closed loop, and the network evidence is shown in Figure 3B.

The results of the inconsistency test revealed the following: HAMD scores involved one closed loop, the lower limit of the 95% CI for the inconsistency factor included 0, and the IF value was small (IF = 3.602), suggesting that the likelihood of inconsistency in the loop was small (*p* > 0.05), and the degree of influence of direct versus indirect comparisons on the results of the overall NMA was small, as shown in Table 4.

**TABLE 4:** Inconsistency of HAMD.

Loop	IF and 95%CI	Z score	P score
CAS-SNRI-Jieyu pill + SNRI-Xiaoyao pill + SNRI	IF = 3.602, 95%CI (0.00, 10.67)	0.999	0.318

Thirty-seven articles reported the post-HAMD scores of different medications for depression, and the results of direct and indirect comparisons in the NMA were tested for consistency (*p* > 0.05), indicating that the overall consistency between direct and indirect comparisons was good, so the consistency model analysis was used. The results of the network meta-analysis showed that out of the 78 two-by-two comparisons, a total of 14 comparisons had statistically significant differences. The HAMD score after CAS treatment was lower than that of Danzhi Xiaoyao Pill + SNRI [WMD = -6.33, 95%CI (-11.09, -1.57)], Jieyu pill [WMD = -4.68, 95%CI (-9.17, -0.19)], Puyu + SNRI [WMD = -4.47, 95%CI (-8.61, -0.34) and Xiaoyao pill + SNRI [WMD = -3.70, 95%CI (-7.34, -0.05)]; the HAMD score after SNRI treatment was lower than that of Danzhi Xiaoyao Pill + SNRI [WMD = -5.02, 95%CI (-8.09, -1.96)], Jieyu pill [WMD = -3.37, 95%CI (-5.99, -0.75)], Puyu + SNRI [WMD = -3.16, 95%CI (-5.12, -1.21)], and Xiaoyao pill + SNRI [WMD = -2.39, 95%CI (-3.93, -0.84)]; the HAMD score after Danzhi Xiaoyao pill + SNRI treatment was higher than that of Jiawei Xiaoyao + CAS [WMD = 4.78, 95%CI (0.73, 8.83)], Jiawei Xiaoyao + SSRI [WMD = 6.51, 95%CI (0.73, 12.30)] and Jieyu pill + SNRI [WMD = 5.82, 95%CI (2.08, 9.57)]; after treatment, the HAMD score of Jieyu pill was higher than that of Jieyu pill + SNRI [WMD = 4.17, 95%CI (0.78, 7.56)]; the HAMD score of Jieyu pill + SNRI was lower than that of Puyu pill + SNRI [WMD = -3.97, 95%CI (-6.87, -1.06)] and Xiaoyao pill + SNRI [WMD = -3.19, 95%CI (-5.74, -0.63)]. The other pairwise comparisons showed no statistical significance (Table 5).

**TABLE 5:** Network meta-analysis of HAMD WMD (95%CI).

Interventions	CAS	SNRI	Anle pill + SSRI	Danzhi Xiaoyao pill + SNRI	Jiawei Xiaoyao	Jiawei Xiaoyao + CAS
CAS	0					
SNRI	−1.31 (−4.95, 2.33)	0				
Anle pill + SSRI	−3.55 (−10.70, 3.61)	−2.24 (−8.81, 4.33)	0			
Danzhi Xiaoyao pill + SNRI	−6.33 (−11.09, −1.57)^*^	−5.02 (−8.09, −1.96)^*^	−2.78 (−10.03, 4.46)	0		
Jiawei Xiaoyao	−5.65 (−12.06, 0.76)	−4.34 (−9.62, 0.94)	−2.10 (−10.53, 6.32)	0.68 (−5.42, 6.79)	0	
Jiawei Xiaoyao + CAS	−1.55 (−6.06, 2.95)	−0.24 (−2.90, 2.41)	1.99 (−5.09, 9.08)	4.78 (0.73, 8.83)^*^	4.09 (−1.81, 10.00)	0
Jiawei Xiaoyao + SSRI	0.18 (−5.93, 6.29)	1.49 (−3.42, 6.40)	3.73 (−4.47, 11.93)	6.51 (0.73, 12.30)^*^	5.83 (−1.38, 13.04)	1.73 (−3.84, 7.31)
Jieyu pill	−4.68 (−9.17, −0.19)^*^	−3.37 (−5.99, −0.75)^*^	−1.13 (−8.20, 5.94)	1.65 (−2.38, 5.69)	0.97 (−4.92, 6.86)	−3.12 (−6.85, 0.60)
Jieyu pill + SNRI	−0.51 (−4.07, 3.05)	0.80 (−1.35, 2.95)	3.04 (−3.17, 9.25)	5.82 (2.08, 9.57)^*^	5.14 (−0.56, 10.84)	1.05 (−2.37, 4.46)
Puyu + SNRI	−4.47 (−8.61, −0.34)^*^	−3.16 (−5.12, −1.21)^*^	−0.93 (−7.78, 5.93)	1.86 (−1.78, 5.50)	1.18 (−4.45, 6.80)	−2.92 (−6.21, 0.37)
Shumian capsule + SSRI	−2.48 (−7.34, 2.38)	−1.17 (−4.39, 2.04)	1.06 (−6.25, 8.38)	3.85 (−0.59, 8.29)	3.17 (−3.01, 9.35)	−0.93 (−5.10, 3.24)
Xiaoyao pill + CAS	−2.72 (−7.28, 1.83)	−1.42 (−4.15, 1.32)	0.82 (−6.29, 7.94)	3.61 (−0.50, 7.72)	2.92 (−3.02, 8.87)	−1.17 (−4.98, 2.64)
Xiaoyao pill + SNRI	−3.70 (−7.34, −0.05)^*^	−2.39 (−3.93, −0.84)^*^	−0.15 (−6.86, 6.57)	2.64 (−0.80, 6.07)	1.95 (−3.54, 7.45)	−2.14 (−5.21, 0.93)

Interventions	Jiawei Xiaoyao + SSRI	Jieyu pill	Jieyu pill + SNRI	Puyu + SNRI	Shumian capsule + SSRI	Xiaoyao pill + CAS	Xiaoyao pill + SSRI
Jiawei Xiaoyao + SSRI	0						
Jieyu pill	−4.86 (−10.42, 0.71)	0					
Jieyu pill + SNRI	−0.69 (−6.05, 4.67)	4.17 (0.78, 7.56)^*^	0				
Puyu + SNRI	−4.65 (−9.94, 0.63)	0.20 (−3.06, 3.47)	−3.97 (−6.87, −1.06)^*^	0			
Shumian capsule + SSRI	−2.66 (−8.53, 3.21)	2.20 (−1.95, 6.34)	−1.98 (−5.84, 1.89)	1.99 (−1.78, 5.76)	0		
Xiaoyao pill + CAS	−2.91 (−8.53, 2.72)	1.95 (−1.84, 5.74)	−2.22 (−5.70, 1.26)	1.75 (−1.62, 5.11)	−0.24 (−4.47, 3.98)	0	
Xiaoyao pill + SNRI	−3.88 (−9.02, 1.27)	0.98 (−2.06, 4.02)	−3.19 (−5.74, −0.63)^*^	0.78 (−1.71, 3.27)	−1.21 (−4.78, 2.36)	−0.97 (−4.12, 2.17)	0

We further ranked all treatments according to SUCRA. The probability ordering results of HAMD score show that Danzhi Xiaoyao pill + SNRI (90.1%) > Jiawei Xiaoyao (79.1%) > Jieyu pill (75.5%) > Puyu + SNRI (74.1%) > Xiaoyao pill + SSRI (63.7%) > Anle pill + SSRI (57.8%) > Xiaoyao pill + CAS(49.4%) > Shumian Capsule + SSRI (46.5%) > Jiawei Xiaoyao + CAS(32.7%) > SNRI (28%) > Jieyu pill + SNRI (18.6%) > Jiawei Xiaoyao + SSRI (17.8%) > CAS(16.7%). According to the SUCRA results, Danzhi Xiaoyao pill + SNRI, Jiawei Xiaoyao, JIeyu pill, Puyu + SNRI were the top four interventions that improved the HAMD score the most, where oral Danzhi Xiaoyao Pill + SNRI may be the most effective intervention to reduce the HAMD score (Table 3).

From the funnel plot of HAMD scores, the points were scattered and more symmetrical, suggesting that the possibility of publication bias was small. Three of the scattered points were located at the bottom of the funnel plot, and 11 scattered points were outside the 95% CI of the funnel plot, suggesting a certain small sample effect (Figure 4B).

### Adverse Effects

The results of the evidence network revealed the following: A total of 32 studies reported the rate of adverse reactions following the use of interventions for depression, involving 12 intervention modalities, and no closed-loop inconsistency tests were required as the medication regimens did not form a closed loop with each other. The network evidence is shown in Figure 3C.

Thirty-two articles reported the adverse effects of different medications for depression, and the results of direct and indirect comparisons in the network meta-analysis were tested for consistency (*p* > 0.05), indicating that the overall consistency between direct and indirect comparisons was good, so the consistency model analysis was used. The results of the network meta-analysis showed that there were 18 statistical differences between the two comparisons, and the rate of adverse reactions after CAS treatment was higher than that of Anle pill + SSRI [RR = 22.40, 95%CI (1.24, 406.26)], Danzhi Xiaoyao pill + SNRI [RR = 22.90, 95%CI (1.18, 445.59)], Jiawei Xiaoyao + SSRI [RR = 18.08, 95%CI (1.11, 293.73)], Jieyu pill [RR = 59.10, 95%CI (3.88, 901.30)], Jieyu pill + SNRI [RR = 17.64, 95%CI (1.09, 285.89)], Puyu + SNRI [20.91, 95%CI (1.23, 356.80)], and Xiaoyao pill + SNRI [RR = 19.02, 95%CI (1.17, 309.37)]; the rate of adverse reactions after SNRI treatment was higher than that of Jiawei Xiaoyao [RR = 1.43, 95%CI (1.00, 2.05)], Jiawei Xiaoyao + SSRI [RR = 1.73, 95%CI (1.26, 2.37)], Jieyu pill [RR = 5.65, 95%CI (3.37, 9.48)], Jieyu pill + SNRI [RR = 1.69, 95%CI (1.26, 2.25)], Puyu + SNRI [RR = 2.00, 95%CI (1.08, 3.69)], and Xiaoyao pill + SNRI [RR = 1.82, 95%CI (1.32, 2.51)]; the adverse reaction rate after Jiawei Xiaoyao + SSRI treatment was higher than that of Jieyu pill [RR = 3.27, 95%CI (1.79, 5.97)]; the adverse reaction rate of Jieyu pill was lower than that of Jieyu pill + SNRI [RR = 0.30, 95%CI (0.17, 0.54)], Puyu + SNRI [RR = 0.35, 95%CI (0.16, 0.79)], Shumian Capsule + SSRI [RR = 0.23, 95%CI (0.10, 0.53)] and Xiaoyao pill + SNRI [RR = 0.32, 95%CI (0.17, 0.59)]. The other pairwise comparisons showed no statistical significance (Table 6).

**TABLE 6:** Network meta-analysis of adverse reactions RR (95%CI).

Interventions	CAS	SNRI	Anle pill + SSRI	Danzhi Xiaoyao + SNRI	Jiawei Xiaoyao	Jiawei Xiaoyao + CAS
CAS	0					
SNRI	10.45 (0.65, 166.89)	0				
Anle pill + SSRI	22.40 (1.24, 406.26)^*^	2.14 (0.92, 5.02)	0			
Danzhi Xiaoyao pill + SNRI	22.90 (1.18, 445.59)^*^	2.19 (0.75, 6.36)	1.02 (0.26, 4.00)	0		
Jiawei Xiaoyao	14.98 (0.92, 244.86)	1.43 (1.00, 2.05)^*^	0.67 (0.27, 1.68)	0.65 (0.21, 2.02)	0	
Jiawei Xiaoyao + CAS	10.45 (0.60, 180.66)	1.00 (0.51, 1.95)	0.47 (0.16, 1.38)	0.46 (0.13, 1.61)	0.70 (0.33, 1.49)	0
Jiawei Xiaoyao + SSRI	18.08 (1.11, 293.73)^*^	1.73 (1.26, 2.37)^*^	0.81 (0.33, 2.00)	0.79 (0.26, 2.40)	1.21 (0.74, 1.96)	1.73 (0.83, 3.62)
Jieyu pill	59.10 (3.88, 901.30)^*^	5.65 (3.37, 9.48)^*^	2.64 (0.98, 7.14)	2.58 (0.79, 8.44)	3.95 (2.10, 7.42)	5.65 (2.43, 13.16)
Jieyu pill + SNRI	17.64 (1.09, 285.89)^*^	1.69 (1.26, 2.25)^*^	0.79 (0.32, 1.93)	0.77 (0.26, 2.33)	1.18 (0.74, 1.87)	1.69 (0.82, 3.49)
Puyu + SNRI	20.91 (1.23, 356.80)^*^	2.00 (1.08, 3.69)^*^	0.93 (0.33, 2.66)	0.91 (0.27, 3.12)	1.40 (0.69, 2.84)	2.00 (0.81, 4.95)
Shumian capsule + SSRI	13.61 (0.79, 233.89)	1.30 (0.69, 2.48)	0.61 (0.21, 1.76)	0.59 (0.17, 2.06)	0.91 (0.43, 1.90)	1.30 (0.52, 3.29)
Xiaoyao pill + SNRI	19.02 (1.17, 309.37)^*^	1.82 (1.32, 2.51)^*^	0.85 (0.34, 2.11)	0.83 (0.27, 2.53)	1.27 (0.79, 2.03)	1.82 (0.87, 3.82)

Interventions	Jiawei Xiaoyao + SSRI	Jieyu pill	Jieyu pill + SNRI	Puyu + SNRI	Shumian capsule + SSRI	Xiaoyao pill + SNRI
Jiawei Xiaoyao + SSRI	0					
Jieyu pill	3.27 (1.79, 5.97)^*^	0				
Jieyu pill + SNRI	0.98 (0.64, 1.50)	0.30 (0.17, 0.54)^*^	0			
Puyu + SNRI	1.16 (0.58, 2.30)	0.35 (0.16, 0.79)^*^	1.19 (0.60, 2.33)	0		
Shumian capsule + SSRI	0.75 (0.37, 1.54)	0.23 (0.10, 0.53)^*^	0.77 (0.38, 1.56)	0.65 (0.27, 1.58)	0	
Xiaoyao pill + SNRI	1.05 (0.66, 1.66)	0.32 (0.17, 0.59)^*^	1.08 (0.70, 1.66)	0.91 (0.46, 1.81)	1.40 (0.68, 2.87)	0

We further ranked all treatments according to SUCRA. The ranking results of adverse reactions showed that Jieyu pill (99.1%) > Anle pill + SSRI (68.9%) > Danzhi Xiaoyao pill + SNRI (67.9%) > Puyu + SNRI (67.6%) > Xiaoyao pill + SSRI (62.9%) > Jiawei Xiaoyao + SSRI (58.5%) > Jieyu pill + SNRI (55.8%) > Jiawei Xiaoyao (42.1%)＞Shumian capsule + SSRI (36.2%) > Jiawei Xiaoyao + CAS(21.8%) > SNRI (16.5%) > CAS(2.7%). According to SUCRA results, Jieyu pill, Anle pill + SSRI, Danzhi Xiaoyao pill + SNRI, Puyu + SNRI were the top four with lower rates of adverse reactions with Jieyu pill being the intervention with the fewest adverse reactions (Table 3).

From the funnel plot of the adverse reaction rates, most of the study scatters were located above the funnel plot with a biased distribution suggesting some publication bias. One scatter was located at the bottom of the funnel plot and one scatter was outside the 95% CI of the funnel plot, suggesting some small sample effect (Figure 4C).

## Results of Network Pharmacology Analysis

### Active Compounds of DZXY

**FIGURE 5:**
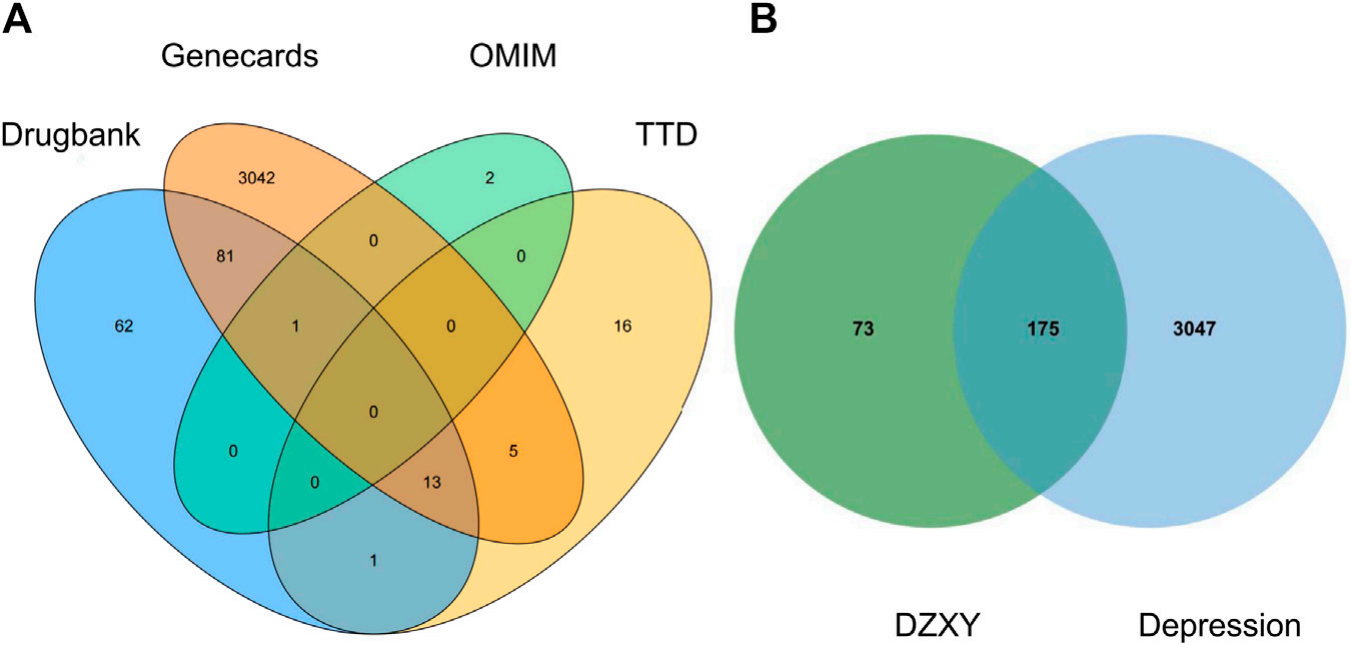
Venn diagrams showing depression targets obtained from four databases **(A)** and the intersection of identified target genes of active compounds and depression **(B)**, respectively.

After searching TCMSP and performing ADME screening (OB ≥ 30 and DL ≥ 0.18), a total of 147 bioactive compounds (*Bupleurum chinense* DC.: 13, *Angelica sinensis* (Oliv.) Diels: 2, *Paeonia lactiflora* Pall.: 8, *Atractylodes macrocephala* Koidz.: 4, *Poria cocos* (Schw.) Wolf.: 6, *Glycyrrhiza uralensis* Fisch. ex DC.: 87, *Paeonia* × suffruticosa Andrews: 6, *Gardenia jasminoides* J. Ellis: 12, *Mentha canadensis* L.: 9) were recognized in DZXY. In addition, depression-related targets were retrieved from four public databases: 3142 genes in GeneCards, 196 in DrugBank, 48 in TTD, and three in OMIM by using “depression” as the keyword. A total of 3,222 related target genes for depression were collected after removing duplication values (Figure 5A). There were 175 overlapping identified targets among DZXY and depression considered as the hub targets for subsequent study (Figure 5B). The names of the components and corresponding targets of DZXY and the specific names of the targets of depression are given in Supplementary Table S2.

### Construction of DZXY Component-Target Network

**FIGURE 6:**
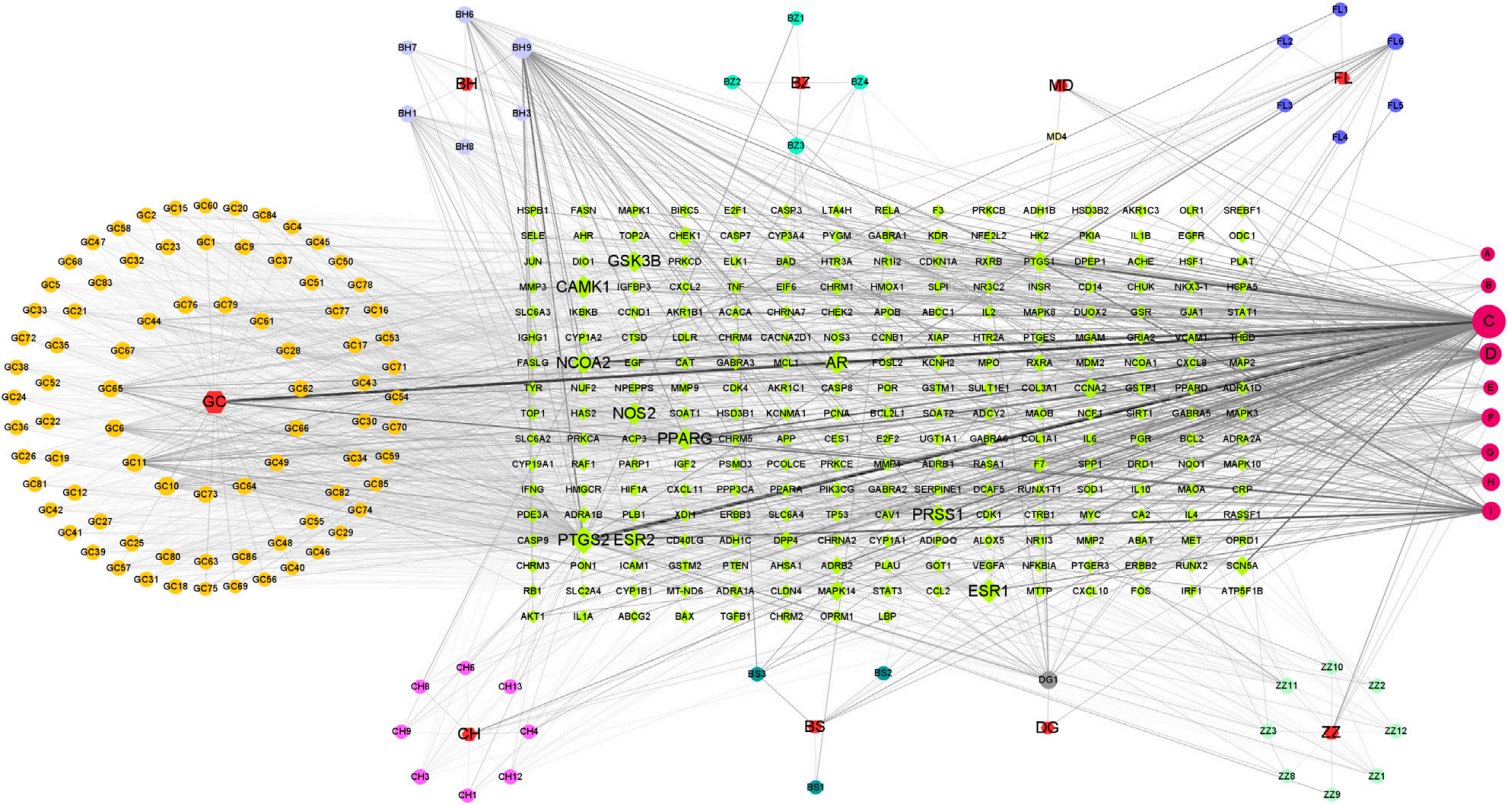
Component-target network of DZXY.

Ingredients and targets, related to nine traditional Chinese medicines of DZXY, were input into Excel tables with the corresponding relationship and properties. Then, the Excel tables were imported into Cytoscape 3.9.0 to make a component-target network. The association between 147 active compounds in DZXY and 248 DZXY target genes was visualized by a component-target network containing 454 nodes and 2,569 edges (Figure 6). Nodes with a greater number of edges have higher degree values and larger node sizes in the network indicating greater significance and requiring more attention. The top five compound nodes with the largest degree size were quercetin (C:MOL000098, n = 149), kaempferol (D:MOL000422, n = 63), luteolin (BH9:MOL005573, n = 56), beta-sitosterol (F:MOL000358, n = 138), 7-methoxy-2-methyl isoflavone (GC10:MOL003896, n = 38). The top five gene nodes with the largest degree size were PTGS2, ESR1, CAMK1, AR, and NOS2, with degree values of 107, 82, 76, 74, and 72, respectively. These top compounds and genes may be the significant nodes in the network and possess an important anti-depression effect.

### Construction of DZXY-Depression-Related PPI Network

**FIGURE 7:**
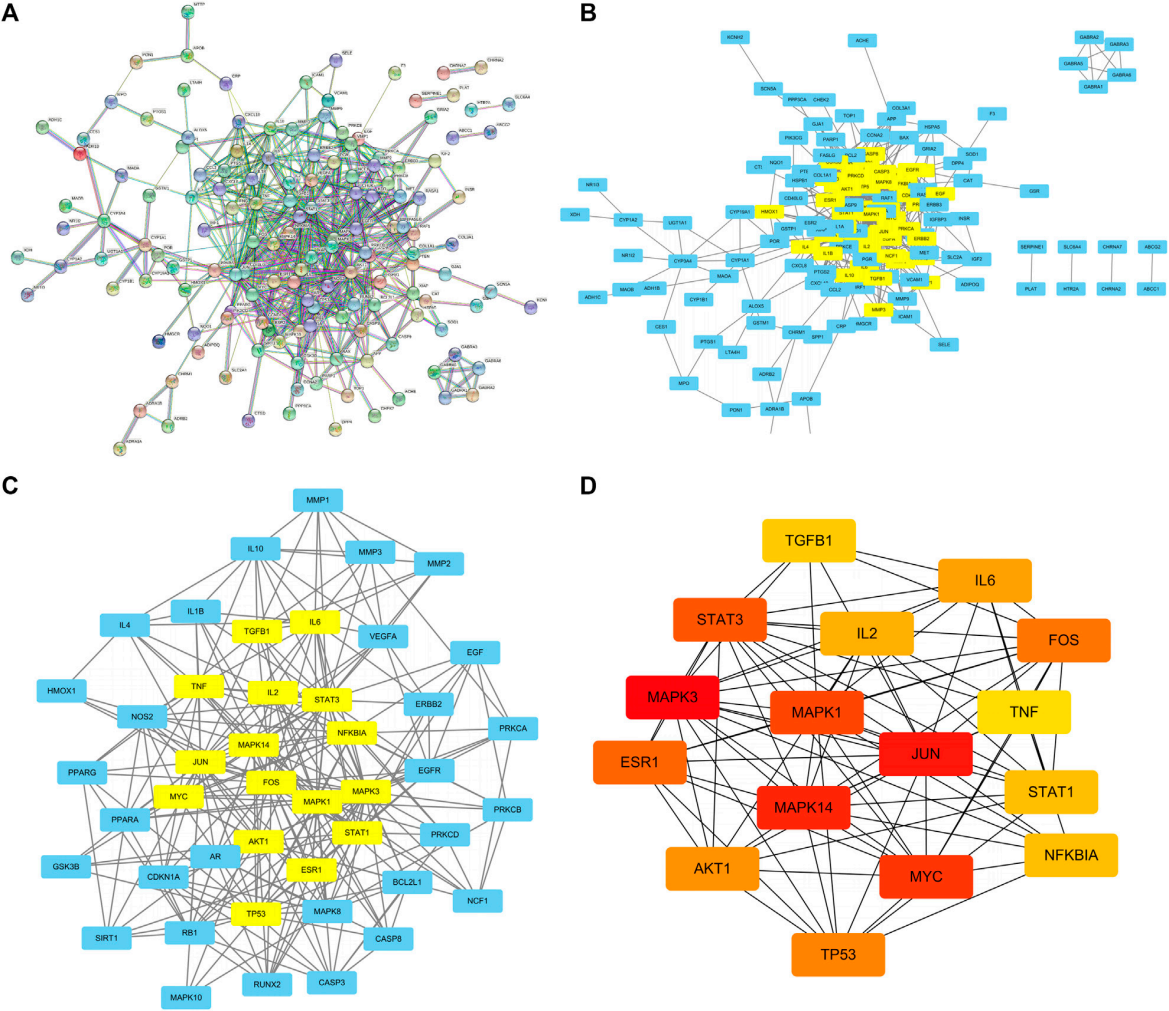
PPI network of DZXY- depression obtained from STRING database **(A)**, PPI network imported from STRING database to Cytoscape 3.9.0 **(B)**, PPI network of core genes by filtering 6 parameters: BC, CC, DC, EC, NC, and LAC **(C)**, and the top 16 MCC gene nodes **(D)**, respectively.

The PPI network which contains 174 nodes and 585 edges was primitively constructed using STRING database, in which nodes represent proteins and edges stand for protein-protein interactions (Figure 7A). Then, the TSV file of the abovementioned PPI was imported into Cytoscape 3.9.0 to construct a new PPI network, which also includes 147 nodes and 585 edges (Figure 7B). Subsequently, numbers of betweenness centrality (BC), closeness centrality (CC), degree centrality (DC), eigenvector centrality (EC), network centrality (NC), and local average connectivity (LAC) greater than corresponding median values were extracted circularly by CytoNCA plugin for mining the core goals (Figure 7C). We selected the top 16 maximal clique centrality (MCC) protein nodes as candidate genes, including MAPK3, JUN, MAPK14, MYC, MAPK1, STAT3, ESR1, FOS, TP53, AKT1, IL6, IL2, NFKBIA, STAT1, TGFB1, and TNF (Figure 7D and Supplementary Table S3), in which color of the nodes represents the intensity of correlation with yellow being the lowest and red being the highest.

### Enrichment Analysis and Component-Target Network

**FIGURE 8:**
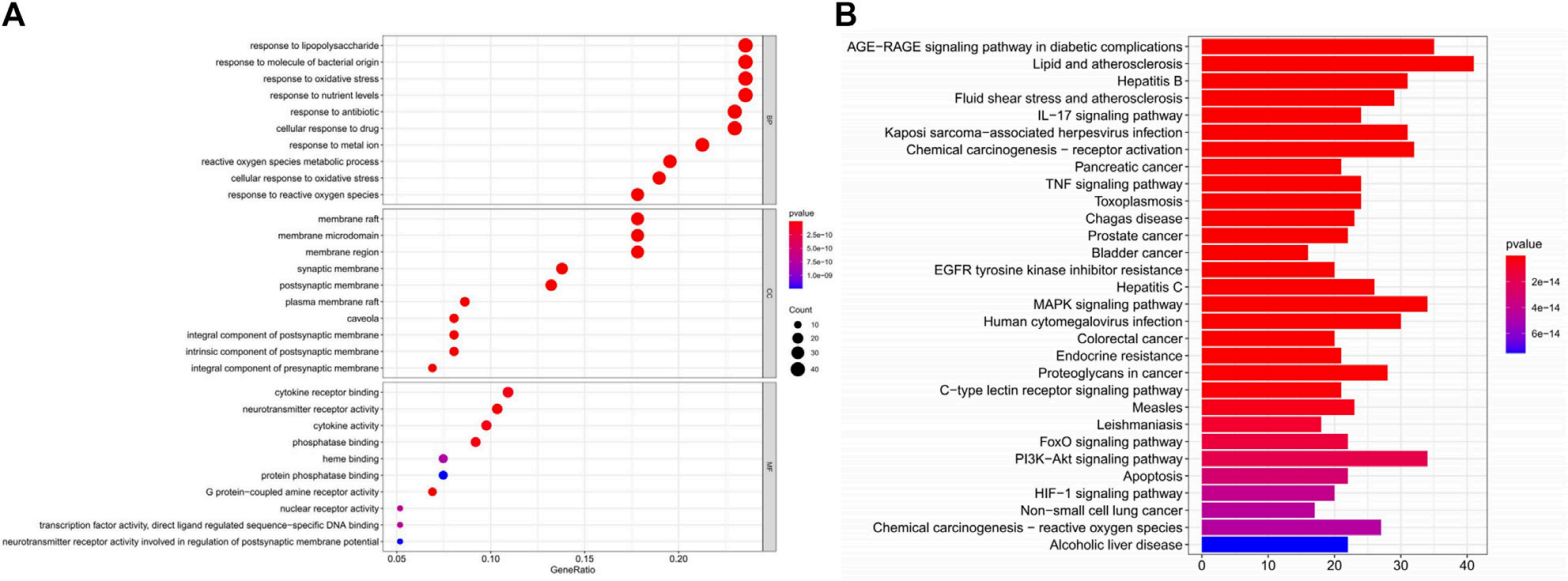
Bubble diagram of GO enrichment analysis of DZXY-Depression genes **(A)** and the bar plot diagram of KEGG enrichment pathways **(B)**, respectively.

**FIGURE 9:**
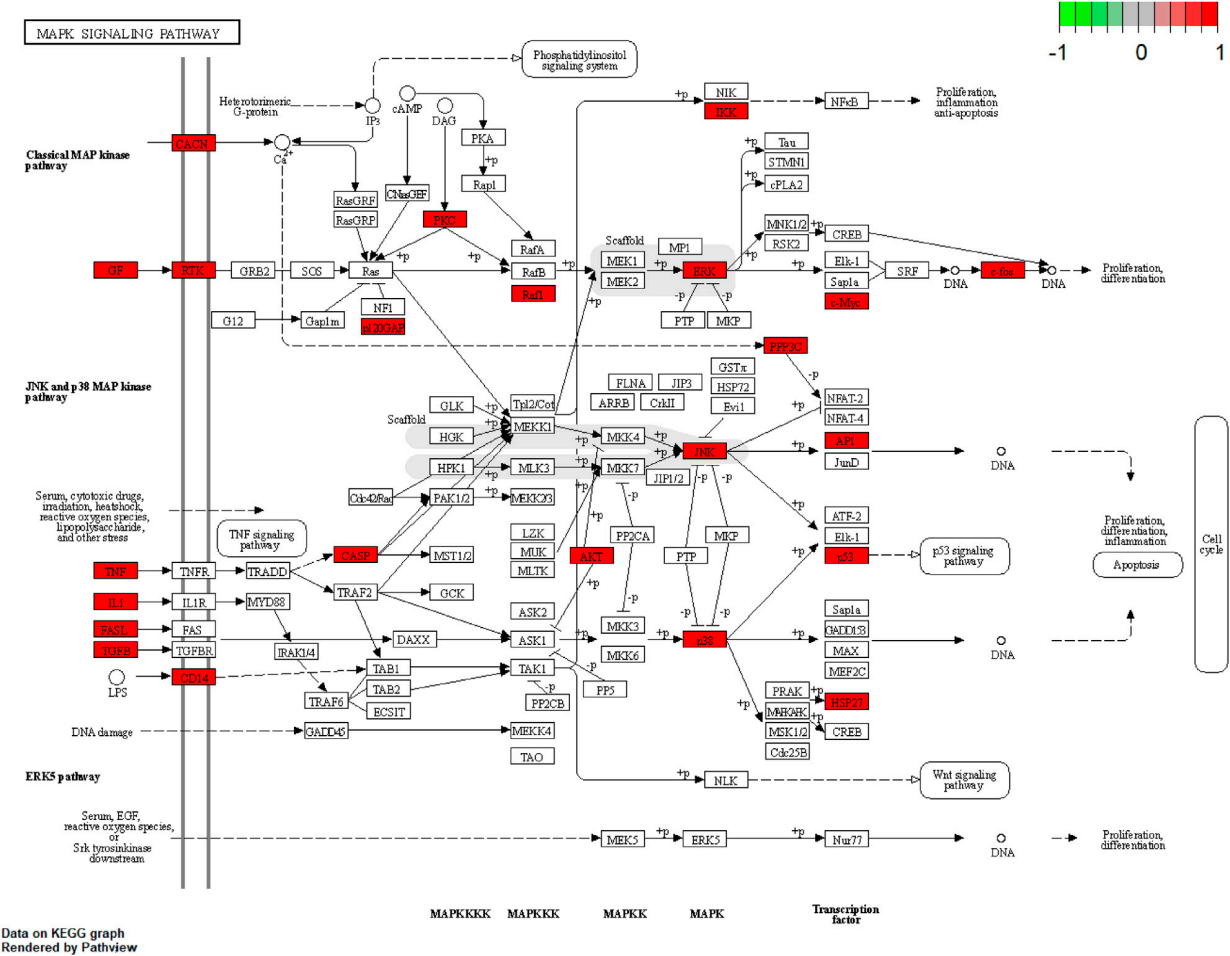
MAPK signaling pathway map.

We performed GO analysis of 175 disease-drug common targets, including biological process (BP), cellular component (CC), and molecular function (MF) using R software (Figure 8A and Supplementary Table S4). The GO results showed that intersecting genes were enriched in 2722 BP pathways. The top six terms in the BP were: response to lipopolysaccharide (GO:0032496), response to molecule of bacterial origin (GO:0002237), response to oxidative stress (GO:0006979), response to nutrient levels (GO:0031667), response to antibiotic (GO:0046677), cellular response to drug (GO:0035690); the intersecting genes were enriched in 144 CC pathways, which were mainly enriched in the membrane raft (GO:0045121), membrane microdomain (GO:0098857), membrane region (GO:0098589), synaptic membrane (GO:0097060), postsynaptic membrane (GO:0045211); the intersecting genes were enriched in 234 MF pathways, which primarily enriched in cytokine receptor binding (GO:0005126), receptor ligand activity (GO:0048018), neurotransmitter receptor activity (GO:0030594), channel activity (GO:0015267), passive transmembrane (GO:0022803); the KEGG analysis displayed that the compounds were mainly enriched in 186 signaling pathways, which included mainly lipid and atherosclerosis (hsa05417), AGE-RAGE signaling pathway in diabetic complications (hsa04933), MAPK signaling pathway (hsa04010), PI3K-Akt signaling pathway (hsa04151), and pathways of neurodegeneration - multiple diseases (hsa05022) (Figure 8B, and Supplementary Table S5). Among them, the MAPK signaling pathway might be the most important DZXY-depression pathway (Figure 9).

## Discussion

The main environmental factor causing depression may be the triggering of brain cell maladaptation after the occurrence of stressful life events (Fernandez et al., 2018). Depression may be related to stress-induced neurovascular pathological changes, and studies have shown that depressive behavior may be related to the loss of the tightly linked transmembrane protein CLDN5 in the nucleus accumbens, altering the integrity of the blood-brain barrier and thus contributing to the transmission of circulating pro-inflammatory cytokines (Dudek et al., 2020). Dopamine (DA) is an important cellular mechanism for shaping behavioral strategies, and dysregulation of the brain reward system, including DA neurons in the ventral tegmental area, is a major contributor to stressful depression-like behavior (Fernandez et al., 2018). The poor physical and mental health status and higher risk of suicide caused by depressive disorders pose a major challenge to health systems and social security in both developed and developing countries. The combination of Chinese and Western medicine treatment can play a synergistic role in increasing the effectiveness and reducing the toxicity, shortening the onset of antidepressants, improving medication compliance, improving quality of life, reducing the risk of relapse and reignition, and decreasing the rate of disability and morbidity and mortality, which can effectively improve the clinical cure rate and safety (Guo et al., 2020). While the HAMD-17 is widely used in clinical studies, two-thirds of depression-related studies apply the scale so the HAMD-17 was selected as the main index to evaluate the efficacy in this study (Bech, 2015).

This study finally included 37 RCTs based on the nadir criteria involving seven oral proprietary Chinese medicines containing Chaihu and covering 13 interventions, mainly proprietary Chinese medicines containing Chaihu alone or in combination with SSRI, SNRI, and CAS. The combination of these three indicators showed that the combination of Chaihu class TCM with antidepressants was generally better than antidepressant treatment, especially Danzhi Xiaoyao pill + SNRI for depression had significant advantages in improving the efficiency, reducing HAMD score and safety, and the treatment with antidepressants alone was inferior to Chaihu class TCM alone or in combination with antidepressants in reducing the incidence of adverse effects, among which the safety and depression scale scores of the cyclic antidepressants were the worst. This may be an important implication for the application of proprietary Chinese medicines containing Chaihu in the treatment of depression, suggesting that clinical workers can combine proprietary Chinese medicines containing Chaihu with SSRI or SNRI in the treatment of depression to improve the therapeutic effect and reduce the adverse effects, and the treatment modality can be preferred to Danzhi Xiaoyao pill + SNRI. Danzhi Xiaoyao pill is better in improving the efficiency of depression and reducing HAMD scores than other Chaihu-based Chinese medicines, which may be related to the ability of *Paeonia* × suffruticosa Andrews to dredge the liver and wind, clear heat and cool blood, and eliminate blood stasis, and the paeonol in *Paeonia* × suffruticosa Andrews has the effects of protecting the cardiovascular system, promoting microcirculation, antibacterial, anti-inflammatory, anti-tumor, antioxidant, anti-metamorphosis effects, and enhancing immunity (Wu G et al., 2019), and *Gardenia jasminoides* J. Ellis can “diarrhea heart fire and remove annoyance and depression,” gardenia glycosides in gardenia *Gardenia jasminoides* J. Ellis have the effects of liver-protecting and choleretic, antihypertensive, analgesic, anti-inflammatory, sedative, hemostatic and anti-swelling (Wang et al., 2015), and paeonol in combination with each other has better power to clear the heat and can play a better therapeutic effect for depression of liver depression and fire.

Moreover, network pharmacology was conducted further to explore the potential mechanism of DZXY against depression. In this study, we screened 147 active components and 248 targets of DZXY and constructed the PPI networks by integrating 175 intersecting targets of DZXY associated with depression. We found that MAPK3, JUN, MAPK14, MYC, MAPK1, etc. could be the most critical potential antidepressant targets of DZXY. Next, we found that the mechanism of DZXY treatment of depression was closely related to response to lipopolysaccharide, response to molecule of bacterial origin, response to oxidative stress, membrane raft, membrane microdomain, membrane region, cytokine receptor binding, receptor-ligand activity, and neurotransmitter receptor activity by GO functional enrichment analysis. Then, we found that the mechanism of DZXY for depression is strongly correlated with lipid and atherosclerosis, AGE-RAGE signaling pathway in diabetic complications, MAPK signaling pathway, PI3K-Akt signaling pathway, and pathways of neurodegeneration in multiple diseases. Based on a network pharmacological analysis of DZXY and relevant literature, we considered that DZXY might exert its antidepressant effects mainly through the regulation of neuroinflammation and cell apoptosis.

The MAPK pathway is one of the most important regulatory pathways in eukaryotic cells, with four subfamilies, ERK1/2, ERK5, JNK, and p38 MAPK. The stress-induced MAPK signaling pathway regulates the expression of multiple pro-inflammatory mediators and apoptotic signals, causing neuronal cell death, and leading to impaired hippocampal function. Evidence suggests that the MAPK pathway is one of the cellular signaling pathways involved in the antidepressant function (Krishna and Narang, 2008; Falcicchia et al., 2020; Fan et al., 2020; Zhang et al., 2020). DZXY has apoptotic targets (CASP3, AKT1, TP53, JUN, MAPK1, etc.) and inflammatory factor targets (IL-2, IL-4, IL-6, VEGFA, NOS3, etc.). Thus, we suggest that DZXY mediates neuroinflammation by regulating inflammatory factors, which is essential for antidepressant effects.

The limitations of this study mainly include the following four points: (i) this study selected week 4 as the time point for judging HAMD scores, and there is a lack of observation for long-term efficacy and whether relapse occurs after drug discontinuation; (ii) the sample size of some of the included literature is small, and the quality evaluation of the literature implemented by allocation concealment and blinding is mostly unclear, which may lead to the potential risk of implementation bias and measurement bias, and the test efficacy is reduced affecting the results of authenticity and reliability; (iii) the included literature studies of the Chaihu class of proprietary Chinese medicines used alone are small, and it is impossible to assess the efficacy advantages and disadvantages between the Chaihu class of prescriptions; (iv) the efficiency and adverse effects of the interventions do not exist in a closed-loop, and the results of mixed comparisons are lacking.

In conclusion, despite the advantages of Chaihu-based proprietary Chinese medicines in improving depression symptoms and safety, the uneven quality of RCTs is not conducive to promoting the internationalization of Chinese medicine. It is strongly recommended that MDD-related studies should be based on the guidelines of objectification, standardization, normalization, and multi-angle, multi-center, and large-sampled CONSORT standard clinical trials to minimize the risk of bias and promote the internationalization of Chinese medicine, with a view to providing a reliable evidence-based theoretical basis for guiding the clinical use of prescriptions.

## Data Availability

The datasets presented in this study can be found in online repositories. The names of the repository/repositories and accession number(s) can be found in the article/Supplementary Material.
