# Supplementary material for: Proprietary Medicines Containing Bupleurum chinense DC. (Chaihu) for Depression: Network Meta-Analysis and Network Pharmacology Prediction
Source: Front Pharmacol. 2022 Apr 6;13:773537. doi: 10.3389/fphar.2022.773537 (PMC9019785; doi:10.3389/fphar.2022.773537)
Supplement: Supplementary file 1 [file DataSheet1.zip › Data Sheet 1/Supplementary Table S1. Network meta-analysis information.docx]

**Supplementary Table S1. Network meta-analysis information**

**Table A│** Final search strategy for CNKI

**Table B│** Final search strategy for Wanfang

**Table C│** Final search strategy for VIP

**Table D** Final search strategy for CBM

**Table E│** Final search strategy for PubMed

**Table F│** Final search strategy for Web of Science

**Table G│** Final search strategy for Cochrane Central Register of Controlled Trials

**Table H│** Final search strategy for Embase

**Table I**│ The ingredients of proprietary medicines used in 37 studies

**Table A│** Final search strategy for CNKI

| **Query** | **Records** |
| --- | --- |
| (SU='抑郁症' OR SU='抑郁障碍' OR TI='抑郁') AND (SU='临床研究' OR SU='临床观察' OR SU='多中心研究' OR SU='临床试验' OR SU='临床分析' OR TI='影响' OR TI='治疗' OR TI='疗效' ) AND (TI='中医' OR TI='中西医' OR TI='中药' TI='多模式' OR TI='胶囊' OR TI='丸' OR TI= '片' OR SU='冲剂' OR TI='液' OR TI='口服液' OR TI='颗粒' OR TI='合剂') NOT (SU='卒中' OR SU='脑梗死' OR SU='中风' OR SU='肿瘤' OR SU='癌' OR SU='围绝经期' OR SU='更年期' OR SU= '功能性消化不良' OR SU='冠心病' OR SU='糖尿病' OR SU='高血压' OR SU='心绞痛' OR SU='室性早搏' OR SU='帕金森' OR SU='癫痫' OR SU='精神分裂症' OR SU='双相情感障碍' OR SU='产后' OR SU='术后' OR SU='慢性阻塞性肺疾病' OR SU='胃炎' OR SU='肠易激' OR SU='血液透析' OR SU='针灸' OR SU='针刺' OR SU='穴位贴敷' OR SU='大鼠' OR SU='小鼠') | 1992 |

**Table B│** Final search strategy for Wanfang

| **Query** | **Records** |
| --- | --- |
| (主题:"抑郁症" OR 主题:"抑郁障碍" OR 题名:"抑郁") AND (主题:"临床研究" OR 主题:"临床观察" OR 主题:"多中心研究" OR 主题:"临床试验" OR 主题:"临床分析" OR 题名:"影响" OR 题名:"治疗" OR 题名:"疗效") AND (题名:"中医" OR 题名:"中西医" OR 题名:"中药" OR 题名:"多模式" OR 题名:"胶囊" OR 题名:"丸" OR 题名:"片" OR 主题:"冲剂" OR 题名:"液" OR 题名:"口服液" OR 题名:"颗粒" OR 题名:"合剂") NOT (主题:"卒中" OR 主题:"脑梗死" OR 主题:"中风" OR 主题:"肿瘤" OR 主题:"癌" OR 主题:"围绝经期" OR 主题:"更年期" OR 主题:"功能性消化不良" OR 主题:"冠心病" OR 主题:"糖尿病" OR 主题:"高血压" OR 主题:"心绞痛" OR 主题:"室性早搏" OR 主题:"帕金森" OR 主题:"癫痫" OR 主题:"精神分裂症" OR 主题:"双相情感障碍" OR 主题:"产后" OR 主题:"术后" OR 主题:"慢性阻塞性肺疾病" OR 主题:"胃炎" OR 主题:"肠易激" OR 主题:"血液透析" OR 主题:"针灸" OR 主题:"针刺" OR 主题:"穴位贴敷" OR 主题:"大鼠" OR 主题:"小鼠") | 2704 |

**Table C│** Final search strategy for VIP

| Query | Records |
| --- | --- |
| M=(抑郁症 OR 抑郁障碍 OR 抑郁) AND M=(临床研究 OR 临床观察 OR 多中心研究 OR 临床试验 OR 临床分析 OR 影响 OR 治疗 OR 疗效) AND T=(中医 OR 中西医 OR 中药 OR 多模式 OR 胶囊 OR 丸 OR 片 OR 冲剂 OR 液 OR 口服液 OR 颗粒 OR 合剂) NOT M=(卒中 OR 脑梗死 OR 中风 OR 肿瘤 OR 癌 OR 围绝经期 OR 更年期 OR 功能性消化不良 OR 冠心病 OR 糖尿病 OR 高血压 OR 心绞痛 OR 室性早搏 OR 帕金森 OR 癫痫 OR 精神分裂症 OR 双相情感障碍 OR 产后 OR 术后 OR 慢性阻塞性肺疾病 OR 胃炎 OR 肠易激 OR 血液透析 OR 针灸 OR 针刺 OR 穴位贴敷 OR 大鼠 OR 小鼠) | 1966 |

**Table D│** Final search strategy for CBM

| **Query** | **Records** |
| --- | --- |
| (( "抑郁症"[标题:智能] OR "抑郁障碍"[标题:智能] OR "抑郁"[标题:智能]) AND( "临床研究"[标题:智能] OR "临床观察"[标题:智能] OR "多中心研究"[标题:智能] OR "临床试验"[标题:智能] OR "临床分析"[标题:智能] OR "影响"[标题:智能] OR "治疗"[标题:智能] OR "疗效"[标题:智能]) AND( "中医"[标题:智能] OR "中西医"[标题:智能] OR "中药"[标题:智能] OR "多模式"[标题:智能] OR "胶囊"[标题:智能] OR "丸"[标题:智能] OR "片"[标题:智能] OR "冲剂"[标题:智能] OR "液"[标题:智能] OR "口服液"[标题:智能] OR "颗粒"[标题:智能] OR "合剂"[标题:智能])) NOT( "卒中"[常用字段:智能] OR "脑梗死"[常用字段:智能] OR "中风"[常用字段:智能] OR "肿瘤"[常用字段:智能] OR "癌"[常用字段:智能] OR "围绝经期"[常用字段:智能] OR "更年期"[常用字段:智能] OR "功能性消化不良"[常用字段:智能] OR "冠心病"[常用字段:智能] OR "糖尿病"[常用字段:智能] OR "高血压"[常用字段:智能] OR "心绞痛"[常用字段:智能] OR "室性早搏"[常用字段:智能] OR "帕金森"[常用字段:智能] OR "癫痫"[常用字段:智能] OR "精神分裂症"[常用字段:智能] OR "双相情感障碍"[常用字段:智能] OR "产后"[常用字段:智能] OR "术后"[常用字段:智能] OR "慢性阻塞性肺疾病"[常用字段:智能] OR "胃炎"[常用字段:智能] OR "肠易激"[常用字段:智能] OR "血液透析"[常用字段:智能] OR "针灸"[常用字段:智能] OR "针刺"[常用字段:智能] OR "穴位贴敷"[常用字段:智能] OR "大鼠"[常用字段:智能] OR "小鼠"[常用字段:智能]) | 1714 |

**Table E│** Final search strategy for PubMed

| **#ID** | **Topic or intervention** | **Query** | **Records** |
| --- | --- | --- | --- |
| #1 | Disease | "Depression"[MeSH Terms] OR "Depressions"[Title/Abstract] OR "Depressive Symptoms"[Title/Abstract] OR "Depressive Symptom"[Title/Abstract] OR "symptom depressive"[Title/Abstract] OR "symptoms depressive"[Title/Abstract] OR "Emotional Depression"[Title/Abstract] OR "depression emotional"[Title/Abstract] | [163,556](https://pubmed.ncbi.nlm.nih.gov/?term="Depression"[MeSH+Terms]+OR+"Depressions"[Title/Abstract]+OR+"Depressive+Symptoms"[Title/Abstract]+OR+"Depressive+Symptom"[Title/Abstract]+OR+"symptom+depressive"[Title/Abstract]+OR+"symptoms+depressive"[Title/Abstract]+OR+"Emotional+Depression"[Title/Abstract]+OR+"depression+emotional"[Title/Abstract]&ac=no&sort=relevance) |
| #2 | Treatment | "[traditional](C:/Users/Yedong/AppData/Local/youdao/dict/Application/8.9.6.0/resultui/html/index.html#/javascript:;) [Chinese](C:/Users/Yedong/AppData/Local/youdao/dict/Application/8.9.6.0/resultui/html/index.html#/javascript:;) [medicine](C:/Users/Yedong/AppData/Local/youdao/dict/Application/8.9.6.0/resultui/html/index.html#/javascript:;)"[Title] OR "integrative medicine"[Title] OR "wan"[Title] OR "pill*"[Title] OR "capsule*"[Title] OR "tablet*"[Title] OR "granule*"[Title] OR "Infusion"[Title] OR "Oral Liquid"[Title] | [114,693](https://pubmed.ncbi.nlm.nih.gov/?term="traditional+Chinese+medicine"[Title]+OR+"integrative+medicine"[Title]+OR+"wan"[Title]+OR+"pill*"[Title]+OR+"capsule*"[Title]+OR+"tablet*"[Title]+OR+"granule*"[Title]+OR+"Infusion"[Title]+OR+"Oral+Liquid"[Title]&ac=no&sort=relevance) |
| #3 | Study design | "Randomized controlled trial"[Publication Type] OR "Controlled Clinical Trial"[Publication Type] OR "Randomized"[Title/Abstract] OR "Randomised"[Title/Abstract] OR "Randomization"[Title/Abstract] OR "Randomisation"[Title/Abstract] OR "randomly"[Title/Abstract] | [1,198,862](https://pubmed.ncbi.nlm.nih.gov/?term="Randomized+controlled+trial"[Publication+Type]+OR+"Controlled+Clinical+Trial"[Publication+Type]+OR+"Randomized"[Title/Abstract]+OR+"Randomised"[Title/Abstract]+OR+"Randomization"[Title/Abstract]+OR+"Randomisation"[Title/Abstract]+OR+"randomly"[Title/Abstract]&ac=no&sort=relevance) |
| #4 | Final query | #1 AND #2 AND #3 | [101](https://pubmed.ncbi.nlm.nih.gov/?term=%231+AND+%232+AND+%233&ac=no&sort=relevance) |

**Table F│** Final search strategy for Web of Science

| **#ID** | **Topic or intervention** | **Query** | **Records** |
| --- | --- | --- | --- |
| #1 | Disease | TS=(Depression) OR TS=(Depressions) OR TS=(Depressive Symptoms) OR TS=(Depressive Symptom) OR TS=(Symptom, Depressive) OR TS=(Symptoms, Depressive) OR TS=(Emotional Depression) OR TS=(depression emotional) | 500,661 |
| #2 | Treatment | TI=(wan*) OR TI=(pill*) OR TI=(capsule*) OR TI=(tablet*) OR TI=(granule*) OR TI=(infusion) OR TI=(Oral Liquid) | 1,251,433 |
| #3 | Study design | TS=(Randomized controlled trial) OR TS=(Controlled Clinical Trial) TS=(Randomized) OR TS=(Randomised) OR TS=(Randomization) OR TS=(Randomisation) OR AB=(Randomized) AB=(Randomization) AB=(Randomisation) AB=(randomly) | 645,251 |
| #4 | Final query | #1 AND #2 AND #3 | 378 |

**Table G│** Final search strategy for Cochrane Central Register of Controlled Trials

| **#ID** | **Topic or intervention** | **Query** | **Records** |
| --- | --- | --- | --- |
| #1 | Disease | MeSH descriptor: [Depression] explode all trees | 12649 |
| #2 |  | ("Depression" OR "Depressions" OR "Depressive Symptoms" OR "Depressive Symptom" OR "Symptom, Depressive" OR "Symptoms, Depressive" OR "Emotional Depression" OR "depression emotional"):ti,ab,kw | 80755 |
| #3 |  | #1 OR #2 | 80755 |
| #4 | Study design | MeSH descriptor: [Random Allocation] explode all trees | 20621 |
| #5 |  | MeSH descriptor: [Randomized Controlled Trial] explode all trees | 119 |
| #6 |  | ("Randomized controlled trial" OR "Controlled Clinical Trial" OR "Randomized" OR "Randomised" OR "Randomization" OR "Randomisation"):ti,ab,kw | 976762 |
| #7 |  | #6 OR #7 OR #8 | 985338 |
| #8 | drug therapy | ("wan*" OR "pill*" OR"capsule*" OR "tablet*" OR "granule*" OR "infusion" OR "Oral Liquid"):ti | 21066 |
| #9 | Final query | #3 AND #7 AND #8 | 382 |

**Table H│** Final search strategy for Embase

| **#ID** | **Query** | **Records** |
| --- | --- | --- |
| #1 | 'depression'/exp | 527,403 |
| #2 | 'depression':ti | 139,284 |
| #3 | 'depressions':ti | 1,658 |
| #4 | 'depressive symptom':ti | 455 |
| #5 | 'depressive symptoms':ti | 15,656 |
| #6 | 'depression emotional':ti | 11 |
| #7 | 'emotional depression':ti | 5 |
| #8 | #16 OR #17 OR #18 OR #19 OR #22 OR #23 OR #33 | 552,112 |
| #9 | 'wan':ti | 564 |
| #10 | 'pill':ti | 6,042 |
| #11 | 'pills':ti | 3,098 |
| #12 | 'capsule':ti | 19,218 |
| #13 | 'capsules':ti | 6,512 |
| #14 | 'tablet':ti | 12,432 |
| #15 | 'tablets':ti | 20,632 |
| #16 | 'granule':ti | 11,110 |
| #17 | 'granules':ti | 10,782 |
| #18 | 'infusion':ti | 56,338 |
| #19 | 'oral liquid':ti | 725 |
| #20 | #9 OR #10 OR #11 OR #12 OR #13 OR #14 OR #15 OR #16 OR #17 OR #18 OR #19 | 145,731 |
| #21 | #8 AND #20 | 1,655 |
| #22 | #21 AND ('clinical article'/de OR 'clinical study'/de OR 'clinical trial'/de OR 'comparative study'/de OR 'controlled clinical trial'/de OR 'controlled study'/de OR 'double blind procedure'/de OR 'major clinical study'/de OR 'multicenter study'/de OR 'randomized controlled trial'/de OR 'randomized controlled trial topic'/de) | 1,006 |

**Table I│**The ingredients of proprietary medicines used in 37 studies

| **Study** | **Species, concentration** |
| --- | --- |
| Wu Y et al. (2019) | Bupleurum chinense DC. [Apiaceae; Bupleuri radix]、Angelica sinensis (Oliv.) Diels [Apiaceae; Angelicae sinensis radix]、Paeonia lactiflora Pall. [Paeoniaceae; Paeoniae radix alba]、Poria cocos (Schw.) Wolf. [Polyporaceae; Poria]、Glycyrrhiza uralensis Fisch. ex DC. [Fabaceae; Glycyrrhizae radix et rhizoma]、Albizia julibrissin Durazz. [Fabaceae;Albiziae cortex]、Curcuma aromatica Salisb. [Zingiberaceae; Curcumae radix]、Polygala tenuifolia Willd. [Polygalaceae; Polygalae radix]、Ziziphus jujube Mill. Var. spinosa (Bunge) Hu ex H. F. Chou[Rhamnaceae; Semen ziziphi spinosae]、Cyperus rotundus L. [Cyperaceae; Cyperi rhizoma] |
| Bai et al. (2020) | Bupleurum chinense DC. [Apiaceae; Bupleuri radix]、Angelica sinensis (Oliv.) Diels [Apiaceae; Angelicae sinensis radix]、Paeonia lactiflora Pall. [Paeoniaceae; Paeoniae radix alba]、Curcuma aromatica Salisb. [Zingiberaceae; Curcumae radix]、Poria cocos (Schw.) Wolf. [Polyporaceae; Poria]、Lilium brownii var. viridulum Baker [Liliaceae; Lilii bulbus]、Albizia julibrissin Durazz. [Fabaceae;Albiziae cortex]、Glycyrrhiza uralensis Fisch. ex DC. [Fabaceae; Glycyrrhizae radix et rhizoma]、Triticum aestivum L. [Poaceae; Fructus tritici levis]、Ziziphus jujuba Mill. [Rhamnaceae; Jujubae fructus] |
| Rui et al. (2019) | Bupleurum chinense DC. [Apiaceae; Bupleuri radix]、Angelica sinensis (Oliv.) Diels [Apiaceae; Angelicae sinensis radix]、Paeonia lactiflora Pall. [Paeoniaceae; Paeoniae radix alba]、Atractylodes macrocephala Koidz. [Asteraceae; Atractylodis macrocephalae rhizoma]、Poria cocos (Schw.) Wolf. [Polyporaceae; Poria]、Glycyrrhiza uralensis Fisch. ex DC. [Fabaceae; Glycyrrhizae radix et rhizoma]、Paeonia × suffruticosa Andrews [Paeoniaceae; Moutan cortex]、Gardenia jasminoides J.Ellis [Rubiaceae; Gardeniae fructus]、Mentha canadensis L. [Lamiaceae; Menthae haplocalycis herba]、Zingiber officinale Roscoe [Zingiberaceae; Zingiberis rhizoma recens] |
| Chi, (2016) | Bupleurum chinense DC. [Apiaceae; Bupleuri radix]、Angelica sinensis (Oliv.) Diels [Apiaceae; Angelicae sinensis radix]、Paeonia lactiflora Pall. [Paeoniaceae; Paeoniae radix alba]、Atractylodes macrocephala Koidz. [Asteraceae; Atractylodis macrocephalae rhizoma]、Poria cocos (Schw.) Wolf. [Polyporaceae; Poria]、Glycyrrhiza uralensis Fisch. ex DC. [Fabaceae; Glycyrrhizae radix et rhizoma]、Mentha canadensis L. [Lamiaceae; Menthae haplocalycis herba]、Zingiber officinale Roscoe [Zingiberaceae; Zingiberis rhizoma recens] |
| Zhang, (2014) | Bupleurum chinense DC. [Apiaceae; Bupleuri radix]、Angelica sinensis (Oliv.) Diels [Apiaceae; Angelicae sinensis radix]、Paeonia lactiflora Pall. [Paeoniaceae; Paeoniae radix alba]、Atractylodes macrocephala Koidz. [Asteraceae; Atractylodis macrocephalae rhizoma]、Poria cocos (Schw.) Wolf. [Polyporaceae; Poria]、Glycyrrhiza uralensis Fisch. ex DC. [Fabaceae; Glycyrrhizae radix et rhizoma]、Paeonia × suffruticosa Andrews [Paeoniaceae; Moutan cortex]、Gardenia jasminoides J.Ellis [Rubiaceae; Gardeniae fructus]、Mentha canadensis L. [Lamiaceae; Menthae haplocalycis herba]、Zingiber officinale Roscoe [Zingiberaceae; Zingiberis rhizoma recens] |
| Xu, (2014) | Bupleurum chinense DC. [Apiaceae; Bupleuri radix]、Angelica sinensis (Oliv.) Diels [Apiaceae; Angelicae sinensis radix]、Paeonia lactiflora Pall. [Paeoniaceae; Paeoniae radix alba]、Atractylodes macrocephala Koidz. [Asteraceae; Atractylodis macrocephalae rhizoma]、Poria cocos (Schw.) Wolf. [Polyporaceae; Poria]、Glycyrrhiza uralensis Fisch. ex DC. [Fabaceae; Glycyrrhizae radix et rhizoma]、Mentha canadensis L. [Lamiaceae; Menthae haplocalycis herba]、Zingiber officinale Roscoe [Zingiberaceae; Zingiberis rhizoma recens] |
| Wang et al. (2014) | Bupleurum chinense DC. [Apiaceae; Bupleuri radix]、Angelica sinensis (Oliv.) Diels [Apiaceae; Angelicae sinensis radix]、Paeonia lactiflora Pall. [Paeoniaceae; Paeoniae radix alba]、Curcuma aromatica Salisb. [Zingiberaceae; Curcumae radix]、Poria cocos (Schw.) Wolf. [Polyporaceae; Poria]、Lilium brownii var. viridulum Baker [Liliaceae; Lilii bulbus]、Albizia julibrissin Durazz. [Fabaceae;Albiziae cortex]、Glycyrrhiza uralensis Fisch. ex DC. [Fabaceae; Glycyrrhizae radix et rhizoma]、Triticum aestivum L. [Poaceae; Fructus tritici levis]、Ziziphus jujuba Mill. [Rhamnaceae; Jujubae fructus] |
| Duan and Yan, (2013) | Bupleurum chinense DC. [Apiaceae; Bupleuri radix]、Angelica sinensis (Oliv.) Diels [Apiaceae; Angelicae sinensis radix]、Paeonia lactiflora Pall. [Paeoniaceae; Paeoniae radix alba]、Curcuma aromatica Salisb. [Zingiberaceae; Curcumae radix]、Poria cocos (Schw.) Wolf. [Polyporaceae; Poria]、Lilium brownii var. viridulum Baker [Liliaceae; Lilii bulbus]、Albizia julibrissin Durazz. [Fabaceae;Albiziae cortex]、Glycyrrhiza uralensis Fisch. ex DC. [Fabaceae; Glycyrrhizae radix et rhizoma]、Triticum aestivum L. [Poaceae; Fructus tritici levis]、Ziziphus jujuba Mill. [Rhamnaceae; Jujubae fructus] |
| Pan, (2013) | Bupleurum chinense DC. [Apiaceae; Bupleuri radix]、Angelica sinensis (Oliv.) Diels [Apiaceae; Angelicae sinensis radix]、Paeonia lactiflora Pall. [Paeoniaceae; Paeoniae radix alba]、Curcuma aromatica Salisb. [Zingiberaceae; Curcumae radix]、Poria cocos (Schw.) Wolf. [Polyporaceae; Poria]、Lilium brownii var. viridulum Baker [Liliaceae; Lilii bulbus]、Albizia julibrissin Durazz. [Fabaceae;Albiziae cortex]、Glycyrrhiza uralensis Fisch. ex DC. [Fabaceae; Glycyrrhizae radix et rhizoma]、Triticum aestivum L. [Poaceae; Fructus tritici levis]、Ziziphus jujuba Mill. [Rhamnaceae; Jujubae fructus] |
| Li, (2012) | Bupleurum chinense DC. [Apiaceae; Bupleuri radix]、Angelica sinensis (Oliv.) Diels [Apiaceae; Angelicae sinensis radix]、Paeonia lactiflora Pall. [Paeoniaceae; Paeoniae radix alba]、Curcuma aromatica Salisb. [Zingiberaceae; Curcumae radix]、Poria cocos (Schw.) Wolf. [Polyporaceae; Poria]、Lilium brownii var. viridulum Baker [Liliaceae; Lilii bulbus]、Albizia julibrissin Durazz. [Fabaceae;Albiziae cortex]、Glycyrrhiza uralensis Fisch. ex DC. [Fabaceae; Glycyrrhizae radix et rhizoma]、Triticum aestivum L. [Poaceae; Fructus tritici levis]、Ziziphus jujuba Mill. [Rhamnaceae; Jujubae fructus] |
| Zhou et al. (2013) | Bupleurum chinense DC. [Apiaceae; Bupleuri radix]、Angelica sinensis (Oliv.) Diels [Apiaceae; Angelicae sinensis radix]、Paeonia lactiflora Pall. [Paeoniaceae; Paeoniae radix alba]、Atractylodes macrocephala Koidz. [Asteraceae; Atractylodis macrocephalae rhizoma]、Poria cocos (Schw.) Wolf. [Polyporaceae; Poria]、Glycyrrhiza uralensis Fisch. ex DC. [Fabaceae; Glycyrrhizae radix et rhizoma]、Paeonia × suffruticosa Andrews [Paeoniaceae; Moutan cortex]、Gardenia jasminoides J.Ellis [Rubiaceae; Gardeniae fructus]、Mentha canadensis L. [Lamiaceae; Menthae haplocalycis herba] |
| Chen, (2014) | Bupleurum chinense DC. [Apiaceae; Bupleuri radix]、Angelica sinensis (Oliv.) Diels [Apiaceae; Angelicae sinensis radix]、Paeonia lactiflora Pall. [Paeoniaceae; Paeoniae radix alba]、Atractylodes macrocephala Koidz. [Asteraceae; Atractylodis macrocephalae rhizoma]、Poria cocos (Schw.) Wolf. [Polyporaceae; Poria]、Glycyrrhiza uralensis Fisch. ex DC. [Fabaceae; Glycyrrhizae radix et rhizoma]、Paeonia × suffruticosa Andrews [Paeoniaceae; Moutan cortex]、Gardenia jasminoides J.Ellis [Rubiaceae; Gardeniae fructus]、Mentha canadensis L. [Lamiaceae; Menthae haplocalycis herba] |
| Zhang and Su, (2009) | Bupleurum chinense DC. [Apiaceae; Bupleuri radix]、Angelica sinensis (Oliv.) Diels [Apiaceae; Angelicae sinensis radix]、Paeonia lactiflora Pall. [Paeoniaceae; Paeoniae radix alba]、Atractylodes macrocephala Koidz. [Asteraceae; Atractylodis macrocephalae rhizoma]、Poria cocos (Schw.) Wolf. [Polyporaceae; Poria]、Mentha canadensis L. [Lamiaceae; Menthae haplocalycis herba]、Zingiber officinale Roscoe [Zingiberaceae; Zingiberis rhizoma recens]、Glycyrrhiza uralensis Fisch. ex DC. [Fabaceae; Glycyrrhizae radix et rhizoma] |
| Yu et al. (2008) | Bupleurum chinense DC. [Apiaceae; Bupleuri radix]、Angelica sinensis (Oliv.) Diels [Apiaceae; Angelicae sinensis radix]、Conioselinum anthriscoides 'Chuanxiong' [Apiaceae; chuanxiong rhizoma]、Poria cocos (Schw.) Wolf. [Polyporaceae; Poria]、Uncaria rhynchophylla (Miq.) Miq. [Rubiaceae; Uncariae ramulus cum uncis]、Reynoutria multiflora (Thunb.) Moldenke [Polygonaceae; Polygoni multiflori caulis]、Atractylodes macrocephala Koidz. [Asteraceae; Atractylodis macrocephalae rhizoma]、Glycyrrhiza uralensis Fisch. ex DC. [Fabaceae; Glycyrrhizae radix et rhizoma] |
| Ma, (2007) | Bupleurum chinense DC. [Apiaceae; Bupleuri radix]、Angelica sinensis (Oliv.) Diels [Apiaceae; Angelicae sinensis radix]、Paeonia lactiflora Pall. [Paeoniaceae; Paeoniae radix alba]、Atractylodes macrocephala Koidz. [Asteraceae; Atractylodis macrocephalae rhizoma]、Poria cocos (Schw.) Wolf. [Polyporaceae; Poria]、Glycyrrhiza uralensis Fisch. ex DC. [Fabaceae; Glycyrrhizae radix et rhizoma]、Mentha canadensis L. [Lamiaceae; Menthae haplocalycis herba]、Zingiber officinale Roscoe [Zingiberaceae; Zingiberis rhizoma recens] |
| Du et al. (2006) | Bupleurum chinense DC. [Apiaceae; Bupleuri radix]、Angelica sinensis (Oliv.) Diels [Apiaceae; Angelicae sinensis radix]、Paeonia lactiflora Pall. [Paeoniaceae; Paeoniae radix alba]、Atractylodes macrocephala Koidz. [Asteraceae; Atractylodis macrocephalae rhizoma]、Poria cocos (Schw.) Wolf. [Polyporaceae; Poria]、Mentha canadensis L. [Lamiaceae; Menthae haplocalycis herba]、Zingiber officinale Roscoe [Zingiberaceae; Zingiberis rhizoma recens]、Glycyrrhiza uralensis Fisch. ex DC. [Fabaceae; Glycyrrhizae radix et rhizoma] |
| Zhang, (2004) | Bupleurum chinense DC. [Apiaceae; Bupleuri radix]、Angelica sinensis (Oliv.) Diels [Apiaceae; Angelicae sinensis radix]、Paeonia lactiflora Pall. [Paeoniaceae; Paeoniae radix alba]、Atractylodes macrocephala Koidz. [Asteraceae; Atractylodis macrocephalae rhizoma]、Poria cocos (Schw.) Wolf. [Polyporaceae; Poria]、Glycyrrhiza uralensis Fisch. ex DC. [Fabaceae; Glycyrrhizae radix et rhizoma]、Mentha canadensis L. [Lamiaceae; Menthae haplocalycis herba]、Zingiber officinale Roscoe [Zingiberaceae; Zingiberis rhizoma recens] |
| Yang et al. (2007) | Bupleurum chinense DC. [Apiaceae; Bupleuri radix]、Angelica sinensis (Oliv.) Diels [Apiaceae; Angelicae sinensis radix]、Paeonia lactiflora Pall. [Paeoniaceae; Paeoniae radix alba]、Atractylodes macrocephala Koidz. [Asteraceae; Atractylodis macrocephalae rhizoma]、Poria cocos (Schw.) Wolf. [Polyporaceae; Poria]、Glycyrrhiza uralensis Fisch. ex DC. [Fabaceae; Glycyrrhizae radix et rhizoma]、Paeonia × suffruticosa Andrews [Paeoniaceae; Moutan cortex]、Gardenia jasminoides J.Ellis [Rubiaceae; Gardeniae fructus]、Mentha canadensis L. [Lamiaceae; Menthae haplocalycis herba] |
| Wang et al. (2010) | Bupleurum chinense DC. [Apiaceae; Bupleuri radix]、Angelica sinensis (Oliv.) Diels [Apiaceae; Angelicae sinensis radix]、Paeonia lactiflora Pall. [Paeoniaceae; Paeoniae radix alba]、Curcuma aromatica Salisb. [Zingiberaceae; Curcumae radix]、Poria cocos (Schw.) Wolf. [Polyporaceae; Poria]、Lilium brownii var. viridulum Baker [Liliaceae; Lilii bulbus]、Albizia julibrissin Durazz. [Fabaceae;Albiziae cortex]、Glycyrrhiza uralensis Fisch. ex DC. [Fabaceae; Glycyrrhizae radix et rhizoma]、Triticum aestivum L. [Poaceae; Fructus tritici levis]、Ziziphus jujuba Mill. [Rhamnaceae; Jujubae fructus] |
| Tao, (2006) | Bupleurum chinense DC. [Apiaceae; Bupleuri radix]、Angelica sinensis (Oliv.) Diels [Apiaceae; Angelicae sinensis radix]、Paeonia lactiflora Pall. [Paeoniaceae; Paeoniae radix alba]、Curcuma aromatica Salisb. [Zingiberaceae; Curcumae radix]、Poria cocos (Schw.) Wolf. [Polyporaceae; Poria]、Lilium brownii var. viridulum Baker [Liliaceae; Lilii bulbus]、Albizia julibrissin Durazz. [Fabaceae;Albiziae cortex]、Glycyrrhiza uralensis Fisch. ex DC. [Fabaceae; Glycyrrhizae radix et rhizoma]、Triticum aestivum L. [Poaceae; Fructus tritici levis]、Ziziphus jujuba Mill. [Rhamnaceae; Jujubae fructus] |
| Li et al. (2008) | Bupleurum chinense DC. [Apiaceae; Bupleuri radix]、Angelica sinensis (Oliv.) Diels [Apiaceae; Angelicae sinensis radix]、Paeonia lactiflora Pall. [Paeoniaceae; Paeoniae radix alba]、Curcuma aromatica Salisb. [Zingiberaceae; Curcumae radix]、Poria cocos (Schw.) Wolf. [Polyporaceae; Poria]、Lilium brownii var. viridulum Baker [Liliaceae; Lilii bulbus]、Albizia julibrissin Durazz. [Fabaceae;Albiziae cortex]、Glycyrrhiza uralensis Fisch. ex DC. [Fabaceae; Glycyrrhizae radix et rhizoma]、Triticum aestivum L. [Poaceae; Fructus tritici levis]、Ziziphus jujuba Mill. [Rhamnaceae; Jujubae fructus] |
| Liu, (2017) | Bupleurum chinense DC. [Apiaceae; Bupleuri radix]、Angelica sinensis (Oliv.) Diels [Apiaceae; Angelicae sinensis radix]、Paeonia lactiflora Pall. [Paeoniaceae; Paeoniae radix alba]、Atractylodes macrocephala Koidz. [Asteraceae; Atractylodis macrocephalae rhizoma]、Poria cocos (Schw.) Wolf. [Polyporaceae; Poria]、Glycyrrhiza uralensis Fisch. ex DC. [Fabaceae; Glycyrrhizae radix et rhizoma]、Mentha canadensis L. [Lamiaceae; Menthae haplocalycis herba]、Zingiber officinale Roscoe [Zingiberaceae; Zingiberis rhizoma recens] |
| Shi, (2015) | Bupleurum chinense DC. [Apiaceae; Bupleuri radix]、Angelica sinensis (Oliv.) Diels [Apiaceae; Angelicae sinensis radix]、Conioselinum anthriscoides 'Chuanxiong' [Apiaceae; chuanxiong rhizoma]、Poria cocos (Schw.) Wolf. [Polyporaceae; Poria]、Uncaria rhynchophylla (Miq.) Miq. [Rubiaceae; Uncariae ramulus cum uncis]、Reynoutria multiflora (Thunb.) Moldenke [Polygonaceae; Polygoni multiflori caulis]、Atractylodes macrocephala Koidz. [Asteraceae; Atractylodis macrocephalae rhizoma]、Glycyrrhiza uralensis Fisch. ex DC. [Fabaceae; Glycyrrhizae radix et rhizoma] |
| Zhang, (2015) | Bupleurum chinense DC. [Apiaceae; Bupleuri radix]、Angelica sinensis (Oliv.) Diels [Apiaceae; Angelicae sinensis radix]、Paeonia lactiflora Pall. [Paeoniaceae; Paeoniae radix alba]、Atractylodes macrocephala Koidz. [Asteraceae; Atractylodis macrocephalae rhizoma]、Poria cocos (Schw.) Wolf. [Polyporaceae; Poria]、Glycyrrhiza uralensis Fisch. ex DC. [Fabaceae; Glycyrrhizae radix et rhizoma]、Mentha canadensis L. [Lamiaceae; Menthae haplocalycis herba]、Zingiber officinale Roscoe [Zingiberaceae; Zingiberis rhizoma recens] |
| Chen, (2015) | Bupleurum chinense DC. [Apiaceae; Bupleuri radix]、Angelica sinensis (Oliv.) Diels [Apiaceae; Angelicae sinensis radix]、Paeonia lactiflora Pall. [Paeoniaceae; Paeoniae radix alba]、Atractylodes macrocephala Koidz. [Asteraceae; Atractylodis macrocephalae rhizoma]、Poria cocos (Schw.) Wolf. [Polyporaceae; Poria]、Glycyrrhiza uralensis Fisch. ex DC. [Fabaceae; Glycyrrhizae radix et rhizoma]、Paeonia × suffruticosa Andrews [Paeoniaceae; Moutan cortex]、Gardenia jasminoides J.Ellis [Rubiaceae; Gardeniae fructus]、Mentha canadensis L. [Lamiaceae; Menthae haplocalycis herba] |
| Cheng Q et al. (2012) | Ziziphus jujube Mill. Var. spinosa (Bunge) Hu ex H. F. Chou[Rhamnaceae; Semen ziziphi spinosae]、Bupleurum chinense DC. [Apiaceae; Bupleuri radix]、Paeonia lactiflora Pall. [Paeoniaceae; Paeoniae radix alba]、Albizia julibrissin Durazz. [Fabaceae; Albiziae flos]、Albizia julibrissin Durazz. [Fabaceae;Albiziae cortex]、Bombyx mori Linnaeus[Bombycidae; Bombyx batryticatus]、Cryptotympa pustulata Fabricius[Cicadidae; Periostracum cicadae]、Juncus effusus L. [Juncaceae; Junci medulla] |
| Chen et al. (2015) | Ziziphus jujube Mill. Var. spinosa (Bunge) Hu ex H. F. Chou[Rhamnaceae; Semen ziziphi spinosae]、Bupleurum chinense DC. [Apiaceae; Bupleuri radix]、Paeonia lactiflora Pall. [Paeoniaceae; Paeoniae radix alba]、Albizia julibrissin Durazz. [Fabaceae; Albiziae flos]、Albizia julibrissin Durazz. [Fabaceae;Albiziae cortex]、Bombyx mori Linnaeus[Bombycidae; Bombyx batryticatus]、Cryptotympa pustulata Fabricius[Cicadidae; Periostracum cicadae]、Juncus effusus L. [Juncaceae; Junci medulla] |
| Cheng S et al. (2012) | Ziziphus jujube Mill. Var. spinosa (Bunge) Hu ex H. F. Chou[Rhamnaceae; Semen ziziphi spinosae]、Bupleurum chinense DC. [Apiaceae; Bupleuri radix]、Paeonia lactiflora Pall. [Paeoniaceae; Paeoniae radix alba]、Albizia julibrissin Durazz. [Fabaceae; Albiziae flos]、Albizia julibrissin Durazz. [Fabaceae;Albiziae cortex]、Bombyx mori Linnaeus[Bombycidae; Bombyx batryticatus]、Cryptotympa pustulata Fabricius[Cicadidae; Periostracum cicadae]、Juncus effusus L. [Juncaceae; Junci medulla] |
| Xu, (2012) | Bupleurum chinense DC. [Apiaceae; Bupleuri radix]、Angelica sinensis (Oliv.) Diels [Apiaceae; Angelicae sinensis radix]、Paeonia lactiflora Pall. [Paeoniaceae; Paeoniae radix alba]、Curcuma aromatica Salisb. [Zingiberaceae; Curcumae radix]、Poria cocos (Schw.) Wolf. [Polyporaceae; Poria]、Lilium brownii var. viridulum Baker [Liliaceae; Lilii bulbus]、Albizia julibrissin Durazz. [Fabaceae;Albiziae cortex]、Glycyrrhiza uralensis Fisch. ex DC. [Fabaceae; Glycyrrhizae radix et rhizoma]、Triticum aestivum L. [Poaceae; Fructus tritici levis]、Ziziphus jujuba Mill. [Rhamnaceae; Jujubae fructus] |
| Shi et al. (2008) | Bupleurum chinense DC. [Apiaceae; Bupleuri radix]、Angelica sinensis (Oliv.) Diels [Apiaceae; Angelicae sinensis radix]、Paeonia lactiflora Pall. [Paeoniaceae; Paeoniae radix alba]、Curcuma aromatica Salisb. [Zingiberaceae; Curcumae radix]、Poria cocos (Schw.) Wolf. [Polyporaceae; Poria]、Lilium brownii var. viridulum Baker [Liliaceae; Lilii bulbus]、Albizia julibrissin Durazz. [Fabaceae;Albiziae cortex]、Glycyrrhiza uralensis Fisch. ex DC. [Fabaceae; Glycyrrhizae radix et rhizoma]、Triticum aestivum L. [Poaceae; Fructus tritici levis]、Ziziphus jujuba Mill. [Rhamnaceae; Jujubae fructus] |
| Yang et al. (2015) | Bupleurum chinense DC. [Apiaceae; Bupleuri radix]、Angelica sinensis (Oliv.) Diels [Apiaceae; Angelicae sinensis radix]、Paeonia lactiflora Pall. [Paeoniaceae; Paeoniae radix alba]、Curcuma aromatica Salisb. [Zingiberaceae; Curcumae radix]、Poria cocos (Schw.) Wolf. [Polyporaceae; Poria]、Lilium brownii var. viridulum Baker [Liliaceae; Lilii bulbus]、Albizia julibrissin Durazz. [Fabaceae;Albiziae cortex]、Glycyrrhiza uralensis Fisch. ex DC. [Fabaceae; Glycyrrhizae radix et rhizoma]、Triticum aestivum L. [Poaceae; Fructus tritici levis]、Ziziphus jujuba Mill. [Rhamnaceae; Jujubae fructus] |
| Yang et al. (2012) | Bupleurum chinense DC. [Apiaceae; Bupleuri radix]、Angelica sinensis (Oliv.) Diels [Apiaceae; Angelicae sinensis radix]、Paeonia lactiflora Pall. [Paeoniaceae; Paeoniae radix alba]、Curcuma aromatica Salisb. [Zingiberaceae; Curcumae radix]、Poria cocos (Schw.) Wolf. [Polyporaceae; Poria]、Lilium brownii var. viridulum Baker [Liliaceae; Lilii bulbus]、Albizia julibrissin Durazz. [Fabaceae;Albiziae cortex]、Glycyrrhiza uralensis Fisch. ex DC. [Fabaceae; Glycyrrhizae radix et rhizoma]、Triticum aestivum L. [Poaceae; Fructus tritici levis]、Ziziphus jujuba Mill. [Rhamnaceae; Jujubae fructus] |
| Xu et al. (2007) | Bupleurum chinense DC. [Apiaceae; Bupleuri radix]、Angelica sinensis (Oliv.) Diels [Apiaceae; Angelicae sinensis radix]、Paeonia lactiflora Pall. [Paeoniaceae; Paeoniae radix alba]、Curcuma aromatica Salisb. [Zingiberaceae; Curcumae radix]、Poria cocos (Schw.) Wolf. [Polyporaceae; Poria]、Lilium brownii var. viridulum Baker [Liliaceae; Lilii bulbus]、Albizia julibrissin Durazz. [Fabaceae;Albiziae cortex]、Glycyrrhiza uralensis Fisch. ex DC. [Fabaceae; Glycyrrhizae radix et rhizoma]、Triticum aestivum L. [Poaceae; Fructus tritici levis]、Ziziphus jujuba Mill. [Rhamnaceae; Jujubae fructus] |
| Lu and Zhao, (2015) | Bupleurum chinense DC. [Apiaceae; Bupleuri radix]、Angelica sinensis (Oliv.) Diels [Apiaceae; Angelicae sinensis radix]、Paeonia lactiflora Pall. [Paeoniaceae; Paeoniae radix alba]、Atractylodes macrocephala Koidz. [Asteraceae; Atractylodis macrocephalae rhizoma]、Poria cocos (Schw.) Wolf. [Polyporaceae; Poria]、Glycyrrhiza uralensis Fisch. ex DC. [Fabaceae; Glycyrrhizae radix et rhizoma]、Paeonia × suffruticosa Andrews [Paeoniaceae; Moutan cortex]、Gardenia jasminoides J.Ellis [Rubiaceae; Gardeniae fructus]、Mentha canadensis L. [Lamiaceae; Menthae haplocalycis herba]、Zingiber officinale Roscoe [Zingiberaceae; Zingiberis rhizoma recens] |
| Li and Tan, (2008) | Bupleurum chinense DC. [Apiaceae; Bupleuri radix]、Angelica sinensis (Oliv.) Diels [Apiaceae; Angelicae sinensis radix]、Paeonia lactiflora Pall. [Paeoniaceae; Paeoniae radix alba]、Atractylodes macrocephala Koidz. [Asteraceae; Atractylodis macrocephalae rhizoma]、Poria cocos (Schw.) Wolf. [Polyporaceae; Poria]、Glycyrrhiza uralensis Fisch. ex DC. [Fabaceae; Glycyrrhizae radix et rhizoma]、Mentha canadensis L. [Lamiaceae; Menthae haplocalycis herba]、Zingiber officinale Roscoe [Zingiberaceae; Zingiberis rhizoma recens] |
| Zhou et al. (2012) | Bupleurum chinense DC. [Apiaceae; Bupleuri radix]、Angelica sinensis (Oliv.) Diels [Apiaceae; Angelicae sinensis radix]、Paeonia lactiflora Pall. [Paeoniaceae; Paeoniae radix alba]、Atractylodes macrocephala Koidz. [Asteraceae; Atractylodis macrocephalae rhizoma]、Poria cocos (Schw.) Wolf. [Polyporaceae; Poria]、Glycyrrhiza uralensis Fisch. ex DC. [Fabaceae; Glycyrrhizae radix et rhizoma]、Mentha canadensis L. [Lamiaceae; Menthae haplocalycis herba]、Zingiber officinale Roscoe [Zingiberaceae; Zingiberis rhizoma recens] |
| Gao, (2013) | Bupleurum chinense DC. [Apiaceae; Bupleuri radix]、Angelica sinensis (Oliv.) Diels [Apiaceae; Angelicae sinensis radix]、Paeonia lactiflora Pall. [Paeoniaceae; Paeoniae radix alba]、Atractylodes macrocephala Koidz. [Asteraceae; Atractylodis macrocephalae rhizoma]、Poria cocos (Schw.) Wolf. [Polyporaceae; Poria]、Glycyrrhiza uralensis Fisch. ex DC. [Fabaceae; Glycyrrhizae radix et rhizoma]、Mentha canadensis L. [Lamiaceae; Menthae haplocalycis herba]、Zingiber officinale Roscoe [Zingiberaceae; Zingiberis rhizoma recens] |
